# Biofilm engineering through c-di-GMP tuning boosts bioleaching efficiency and arsenic tolerance in *Acidithiobacillus ferrooxidans*

**DOI:** 10.1128/aem.02288-25

**Published:** 2026-02-18

**Authors:** Xi Han, Yidan Hu, Yanbo Yue, Yuefei Ding, Bin Cao, Liang Shi, Juan Liu

**Affiliations:** 1 The Key Laboratory of Water and Sediment Sciences, College of Environmental Sciences and Engineering, Peking University 12465, Beijing, China; 2 Department of Biological Sciences and Technology, School of Environmental Studies, China University of Geosciences 12564, Wuhan, China; 3 Gene and Linda Voiland School of Chemical Engineering and Bioengineering, Washington State University 6760, , Pullman, Washington, USA; 4 State Key Laboratory of Geomicrobiology and Environmental Changes, China University of Geosciences 12564, Wuhan, China; Colorado School of Mines, Golden, Colorado, USA

**Keywords:** *Acidithiobacillus ferrooxidans*, biofilm matrix, arsenic resistance, bioleaching, genetic engineering, bis(3'-5')-cyclic dimeric guanosine monophosphate (c-di-GMP)

## Abstract

Bioleaching offers a sustainable alternative to conventional metallurgy, but its application is limited by low leaching rates, inhibition by heavy metals, and prolonged adaptation. Here, we engineered *Acidithiobacillus ferrooxidans*, a model bioleaching microorganism ubiquitous in mining environments, by modulating intracellular bis(3′−5′)-cyclic dimeric guanosine monophosphate (c-di-GMP) signaling to enhance biofilm formation, bioleaching efficiency, and arsenic tolerance. Overexpression of diguanylate cyclase genes *AFE_1379*, *AFE_0053*, and *AFE_1373* produced engineered strains S-222, S-306, and S-651, respectively, with 1.7-, 2.5-, and 5-fold higher intracellular c-di-GMP levels than the control carrying the empty plasmid vector. Under arsenic-free conditions, all engineered strains showed similar growth profiles, but S-306, at intermediate c-di-GMP (306.3 ± 28.1 μg mg^−1^), formed cytochrome-rich biofilms with low internal resistance and achieved the highest bioleaching efficiency. Under arsenic stress, S-651, at high c-di-GMP (651.4 ± 15.5 μg mg^−1^), developed polysaccharide-rich biofilms that enhanced arsenic tolerance, scorodite (FeAsO₄·2H₂O) precipitation, and bioleaching performance. Transcriptomic analysis confirmed these strain-specific gene expression patterns. These findings demonstrate that tuning intracellular c-di-GMP enables *A. ferrooxidans* to reprogram biofilm matrix composition for extracellular electron uptake and heavy-metal resistance, providing a synthetic biology strategy for environmentally friendly bioleaching and tailings recycling.

**IMPORTANCE:**

As a model microorganism for bioleaching, *Acidithiobacillus ferrooxidans* is limited in leaching efficiency by several key constraints, including slow biofilm formation and susceptibility to environmental heavy metals. Although genetic engineering has been widely used to tackle these challenges, conventional strategies typically focus on modifying one single trait at a time, which significantly restricts their industrial applicability. In this study, we present a novel approach that overcomes this limitation through targeted modulation of the global regulatory molecule c-di-GMP. Engineering this upstream signaling pathway allowed for the tunable enhancement of both bioleaching efficiency and heavy metal resistance, providing an integrated strategy to address multiple bottlenecks simultaneously. This work offers a versatile and practical biotechnology route for diverse scenarios to enhance bioleaching performance and environmental adaptability, which may facilitate the utilization of low-grade ores and mining tailings and ultimately contribute to more sustainable and circular metal production.

## INTRODUCTION

Bioleaching is a sustainable and eco-friendly biotechnology that utilizes microorganisms to extract valuable metals from low-grade ores and mine tailings (1). In contrast to traditional extraction techniques, such as hydrometallurgy and pyrometallurgy, bioleaching operates at significantly lower energy inputs, generates minimal hazardous emissions, and offers a cost-effective alternative (2, 3). Importantly, its applicability extends beyond primary ores to the recovery of valuable metals from secondary sources, including mine tailings, sewage sludge, and electronic wastes, thereby enabling simultaneous waste management and resource recovery (4, 5). Thus, bioleaching aligns with the needs for reducing carbon footprints and promoting circular economy principles by transforming waste streams into marketable products. Currently, bioleaching contributes approximately 10–20% of global copper production and around 5% of gold (6, 7). With increasingly stringent environmental regulations, growing demand for sustainable resource management, and advances in microbial process engineering, the contribution of bioleaching to global metal supply is expected to expand significantly in the coming decades (8).

Microorganisms commonly employed in bioleaching are acidophilic bacteria and archaea that are capable of catalyzing the oxidation of sulfide minerals (7), including pyrite (FeS_2_), chalcopyrite (CuFeS_2_), and arsenopyrite (FeAsS), to release valuable metals such as gold and copper from ores (9). Among these, *Acidithiobacillus ferrooxidans*, an acidophilic, chemolithoautotrophic sulfur- and iron-oxidizing bacterium, is widely used in bioleaching (2). In particular, its autotrophic metabolism enables growth and efficient metal recovery in environments with extremely low organic contents, such as mine tailings and industrial heaps (10). *A. ferrooxidans* also exhibits relatively high tolerance to acidity and metal-contaminated conditions compared with many microorganisms (11). Nevertheless, its activity can still be inhibited by the exceptionally high metal loads encountered in industrial biomining operations (e.g., arsenite concentrations can reach the 6 g L⁻¹ level in some process solutions) (10, 11). Besides, industrial-scale applications of *A. ferrooxidans* for bioleaching face significant challenges, including slow leaching rates for refractory ores, long startup and microbial adaptation periods, and the need for careful operational control to maintain optimal microbial activity (12). For example, the release of arsenic from arsenopyrite and other arsenic-bearing minerals often occurs during bioleaching, which can induce cellular stress and irreversibly inhibit microbial metabolism (13). The formation of biofilms, in which cells are embedded within extracellular polymeric substances (EPS), can mitigate these limitations by increasing the population of metabolically active cells involved in iron and sulfur oxidation (14), reducing heavy metal toxicity through effective immobilization within the EPS matrix (15–17), and enhancing microbial tolerance to environmental fluctuations (18–20). Therefore, promoting the formation of *A. ferrooxidans* biofilms with optimal structural and functional properties on mineral surfaces represents a crucial strategy to improve the efficiency and sustainability of metal extraction processes.

With the rapid advancement of genetic engineering, increasing efforts have been made to optimize the metabolic or physiological properties of microorganisms used in bioleaching processes (21). For *A. ferrooxidans*, enhancing its iron and sulfur metabolism is a common genetic engineering approach to improve bioleaching efficiency, which can be achieved, for instance, by overexpressing the *rus* gene or by editing sulfur metabolism-related genes (e.g., *tetH* and *sdo*) (22–24). Another practical strategy for *Acidithiobacillus* species is regulating functional genes responsive to low pH, heavy metals, and other environmental stresses (11). Furthermore, the leaching efficiency of these strains can be elevated by promoting EPS biosynthesis and biofilm formation, for example, through adding precursors (e.g., D-galactose) or overexpressing quorum sensing (QS) genes (e.g., *afeI/R*) (25). However, QS-based strategies are not universally applicable, as canonical QS systems are absent in key bioleaching species such as *Acidithiobacillus caldus* and *Acidithiobacillus thiooxidans* (26, 27). In contrast, the bacterial second messenger bis(3′−5′)-cyclic dimeric guanosine monophosphate (c-di-GMP) is conserved across all major *Acidithiobacillus* species (28–31), making it more likely to serve as a genetic engineering target that is applicable to diverse bioleaching microorganisms. Notably, elevated c-di-GMP levels in biofilm cells compared to planktonic cells suggest that increasing intracellular c-di-GMP may enhance the attachment and biofilm formation of bioleaching microbes on mineral surfaces, thereby improving bioleaching efficiency (32). While no direct evidence links c-di-GMP to the expression of Fe- or S-oxidation enzymes in *A. ferrooxidans*, a recent study in dissimilatory iron-reducing bacteria shows that elevated c-di-GMP boosts *c*-type cytochrome expression, enhancing electron transfer at cell-mineral interfaces (33). Thus, in addition to promoting biofilm formation, engineering the c-di-GMP network may also promote electron transfer between *A. ferrooxidans* and extracellular minerals, though such genetic manipulation has yet to be reported in this species.

C-di-GMP levels are dynamically regulated by diguanylate cyclases (DGCs) for synthesis and phosphodiesterases (PDEs) for degradation. In this study, we selected all four endogenous DGC genes previously identified in *A. ferrooxidans* (*AFE_1360*, *AFE_1373*, *AFE_0053*, and *AFE_1379*) (28), which alter c-di-GMP levels upon heterologous expression in *E. coli*, along with one exogenous DGC gene (*yedQ*) from *E. coli* that has been shown to effectively remodel biofilm architecture via c-di-GMP modulation (34). Differences in intracellular c-di-GMP concentrations across these engineered strains were then assessed, along with variations in their abilities for pyrite oxidation, biofilm formation, EPS composition, and arsenic stress tolerance. Our findings revealed that transferring different endogenous DGC genes can elevate c-di-GMP levels in *A. ferrooxidans* to distinct extents, thereby enabling precise regulation of biofilm composition and its efficiencies in extracellular electron transfer and arsenic resistance.

## MATERIALS AND METHODS

### Bacterial strains and culture conditions

Strains, plasmids, and primers used in this study are listed in Supplementary Information (SI) Tables S1 and S2. The wild-type (WT) *A. ferrooxidans* strain ATCC 23270 (purchased from ATCC) was used to construct engineered strains. *E. coli* strains were cultured in Luria-Bertani broth (LB) at 37°C with shaking at 180 rpm. *A. ferrooxidans* WT and its derivative strains were cultured at 30°C with shaking at 180 rpm in 9K-Fe medium supplemented with 62 mM FeSO₄·7H₂O, or 9K-S medium containing 1% (wt/vol) elemental sulfur (35), with detailed medium compositions provided in Table S3. Streptomycin was added to the engineered strain culture at a final concentration of 200 μg mL⁻¹.

### Construction of plasmid and engineered strains

The plasmid pYDT used in this study was constructed based on the plasmid pYYDT, with detailed procedures provided in Section S1 (36). Genes *AFE-1379*, *AFE-0053*, *AFE-1373* (from *A. ferrooxidans* ATCC 23270), and *yedQ* (from *E. coli* BL21), each with an additional ribosome binding site (RBS), were cloned by polymerase chain reaction (PCR) using specific primers (Table S2). These amplified fragments were inserted into pYDT to generate pYDT-1379, pYDT-0053, pYDT-1373, and pYDT-yedQ, with all target genes under the P_tac_ promoter. The constructs were validated by colony PCR using primers YD-F/R and DNA sequencing. Plasmid constructions were conducted in *E. coli* SM10 via heat shock. Plasmids pYDT, pYDT-yedQ, pYDT-1379, pYDT-0053, and pYDT-1373 were introduced into *A. ferrooxidans* by conjugative transfer (35), yielding strains S-137, S-149, S-222, S-306, and S-651. Plasmid isolation and PCR product purification kits were from Thermo Fisher Scientific (Waltham, MA, USA). The plasmid DNA sequence was confirmed by Sanger sequencing after purification from the recombinant strain. The presence of pYDT and its derivatives in *A. ferrooxidans* cells was monitored by extracting plasmids for gel electrophoresis analysis following our experiments.

### Quantification of c-di-GMP

Intracellular c-di-GMP levels were determined according to an established method (37). Briefly, 1 mL of culture was harvested during the late logarithmic growth phase (OD₆₀₀ ≈ 0.4), centrifuged at 16,000 × *g* for 2 min at 4°C, and the pellets were washed twice with 1 mL of ice-cold phosphate-buffered saline (PBS, pH 7.4). Then, cells were resuspended in 1 mL ice-cold PBS and lysed on ice using an ultrasonic disruptor (JY92-IIN, Ningbo Scientz Biotechnology, China) with 3 s pulses with 5 s intervals for a total of 5 min at 60% amplitude. After clearing debris by centrifugation (12,000 × *g*, 5 min, 4°C), the supernatant was collected for c-di-GMP measurements using a commercial enzyme-linked immunosorbent assay (ELISA) kit (Microbial c-di-GMP ELISA Kit, Cat. No. MM-91036O2, Jiangsu Meimian Industrial Co., Ltd., China). Absorbance was measured at 450 nm on a UV-Vis spectrophotometer (UV-2600i, Shimadzu, Japan).

### Microbial oxidation of pyrite

Pyrite used in this study was obtained from a mine in Anhui, China, with powder X-ray diffraction (XRD) confirming that its crystalline phase consisted solely of pyrite (Fig. S1). For XRD analysis, dried particles were mounted on monocrystalline silicon sample holders and measured using a Bruker D8 Advance X-ray diffractometer (Germany) with Cu Kα (40 kV and 40 mA). XRD patterns were recorded from 10˚ to 90˚(2θ) with a 0.015˚ step size and a scan time of 0.2 s per step. The pyrite was ground using an agate mortar, sieved through 500 mesh, and soaked in 1 M hydrochloric acid overnight to remove surface oxidation layers. Washed pyrite was transferred to an anaerobic glovebox (Vigor Laboratory; N₂ atmosphere, <1 ppm O_2_) and sterilized via three cycles of 30-minute immersions in 75% (vol/vol) ethanol, followed by triple rinsing with degassed sterile Milli-Q water. *A. ferrooxidans* cultures were pre-grown in 9K-S medium for 7 days and then processed via sequential centrifugation: sulfur particles were pelleted at 1,500 rpm for 1 min, and bacterial cells were harvested at 8,000 rpm for 5 min. The cell pellet was washed twice with sulfur-free 9K basal salts medium (pH 2) and resuspended in 9K medium containing 5% (wt/vol) pretreated pyrite to achieve an initial OD₆₀₀ of 0.10 ± 0.02. Bioleaching systems (50 mL slurry in 150 mL cone flasks) were incubated statically at 30°C. For arsenic-stressed systems, sodium arsenite (NaAsO₂, >99%, Sigma-Aldrich) was filter-sterilized and added to a final concentration of 5 mM prior to inoculation. At defined intervals, 200 μL aliquots were withdrawn and analyzed for Fe²^+^ using ferrozine assay (38) and sulfate using ion chromatography (ICS-1100, Thermo Fisher Scientific, USA), with detailed procedures provided in Section S2.

### Biofilm characterization

Biofilms formed on pyrite-coated glass slides were analyzed in terms of (i) confocal microscopy, (ii) total protein content, (iii) exopolysaccharide content, and (iv) cytochrome content. Pyrite-coated glass slides were prepared as described previously (39) and inoculated in 9K medium (pH 2.0 ± 0.2) with *A. ferrooxidans* at an initial OD₆₀₀ of 0.10 ± 0.01 to facilitate biofilm growth. The methodologies for quantifying total biofilm protein, exopolysaccharides, and cytochromes are detailed in Section S3. Pyrite-coated glass slides biofilms were imaged under a confocal laser scanning microscope (CLSM, TCS SP8, Leica, Germany), with optimized excitation/emission settings for SYTO 9 (488/550 nm), PI (535/617 nm), and ConA-TRITC (557/576 nm), with detailed procedures provided in Section S3. To investigate how EPS compositions in *A. ferrooxidans* biofilms affect arsenic immobilization, EPS from different engineered strains was extracted, and its arsenic adsorption capability was assessed. Detailed procedures for EPS extraction and arsenic adsorption experiments are provided in Section S4.

### Transcriptome and gene expression analysis of biofilm cells

Biofilm cells during the mid-late stage (day 30) of microbial oxidation were collected for RNA sequencing (Majorbio Co., Ltd., Shanghai, China) and qPCR analysis. Total RNA was extracted using the CTAB method, followed by genomic DNA removal (40). High-quality RNA samples were depleted of ribosomal RNA using the RiboCop rRNA Depletion Kit (Lexogen, USA) to optimize bacterial transcriptome profiling. The mRNA was fragmented into ~200 nt fragments using fragmentation buffer, and sequencing libraries were constructed and sequenced on the Illumina NovaSeq XPlus platform. Sequence reads were mapped onto the *A. ferrooxidans* reference genome (accession no. NC_002939.5) using Hisat2. For targeted gene expression analysis, total RNA was extracted from the biofilm cells collected at day 30 using MiniBEST Universal RNA Extraction Kit (TaKaRa, Japan) and reverse-transcribed to cDNA using the PrimeScript RT reagent Kit (TaKaRa, Japan). DNA concentration was measured with a Nanodrop spectrophotometer. Relative quantification of target gene expression was performed using *alaS* as the reference gene (41), with qPCR reactions amplified on a LightCycler 96 (Roche, Germany). All qPCR primers are listed in Table S2.

## RESULTS AND DISCUSSION

### Construction of engineered *A. ferrooxidans* strains with varying c-di-GMP levels

**Fig 1:**
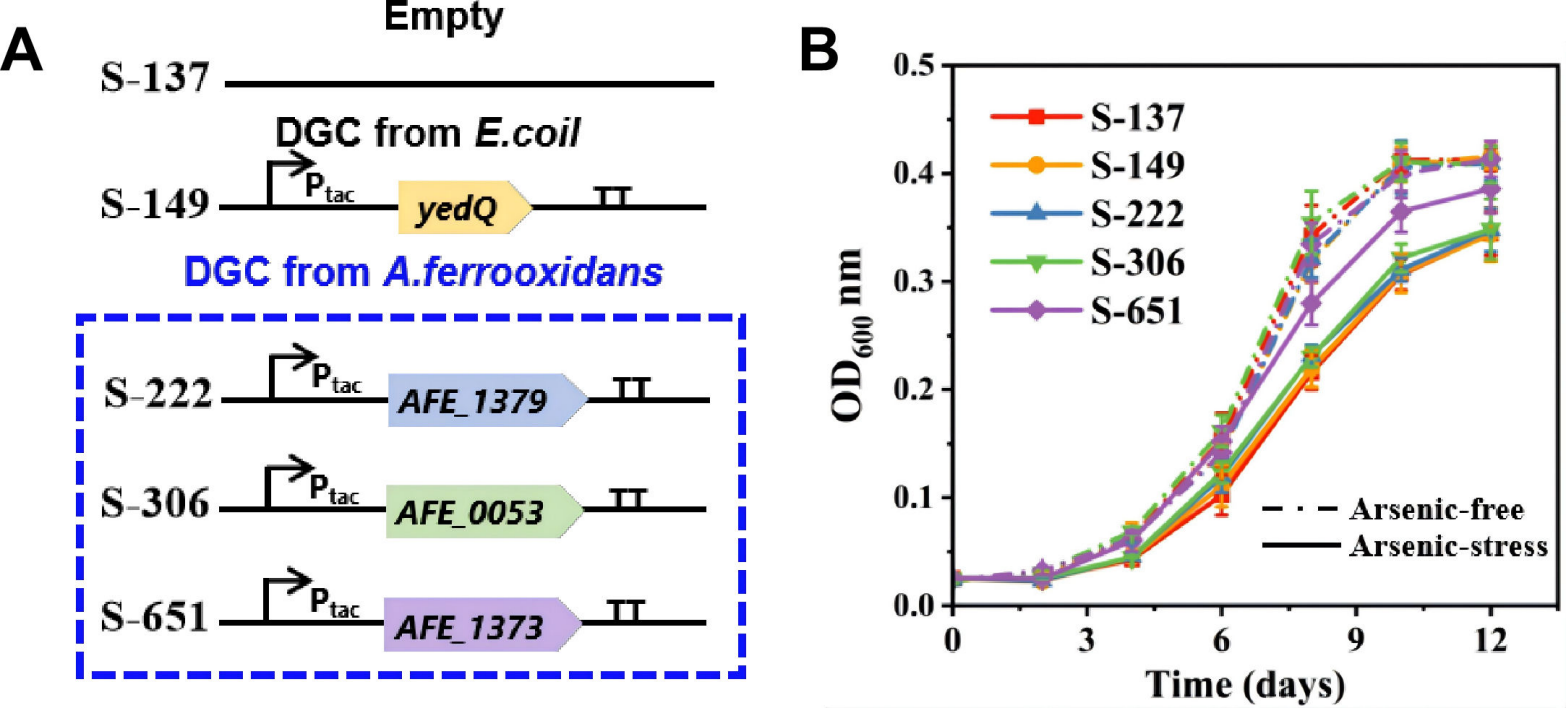
(**A**) Strain designations: S-149 expresses the diguanylate cyclase (DGC) gene *yedQ* (from *E. coli*); S-222, S-306, and S-651 express DGC genes *AFE_1379, AFE_0053*, and *AFE_1373* (from *A. ferrooxidans*), respectively. All DGC genes are under the control of the P_tac_ promoter. (**B**) Growth curves of these strains (with elemental sulfur as the electron donor) under arsenic-free (dashed curves) and arsenic-stressed (solid curves) conditions. Data are represented as mean values ± standard deviation (SD) (*n* = 3 independent replicates).

Five DGC genes, including all four DGC genes annotated in *A. ferrooxidans* (*AFE_1360*, *AFE_1373, AFE_0053*, and *AFE_1379*) (29) and one from *E. coli* (*yedQ*) (34), were cloned into the broad-host plasmid pYDT (Fig. S2). Except for *AFE_1360*, which resulted in non-viable engineered *A. ferrooxidans* strains, all genes were successfully introduced, yielding recombinant plasmids pYDT-1373, pYDT-0053, pYDT-1379, and pYDT-yedQ. Thus, the selected endogenous DGCs comprise all DGCs annotated in the *A. ferrooxidans* genome used in this study, and *AFE_1360* was not included in subsequent phenotypic analyses because its overexpression consistently yielded non-viable strains. The design of the genetic circuit is shown in Fig. 1A. Transformation of these plasmids into *A. ferrooxidans* generated four engineered strains, i.e*.,* S-149 (pYDT-yedQ), S-222 (pYDT-1379), S-306 (pYDT-0053), and S-651 (pYDT-1373), along with a control strain, S-137, carrying the empty pYDT plasmid. These strains displayed comparable growth rates under arsenic-free conditions (Fig. 1B), suggesting that the transformation of these plasmids had no significant impact on the growth of *A. ferrooxidans*.

**Fig 2:**
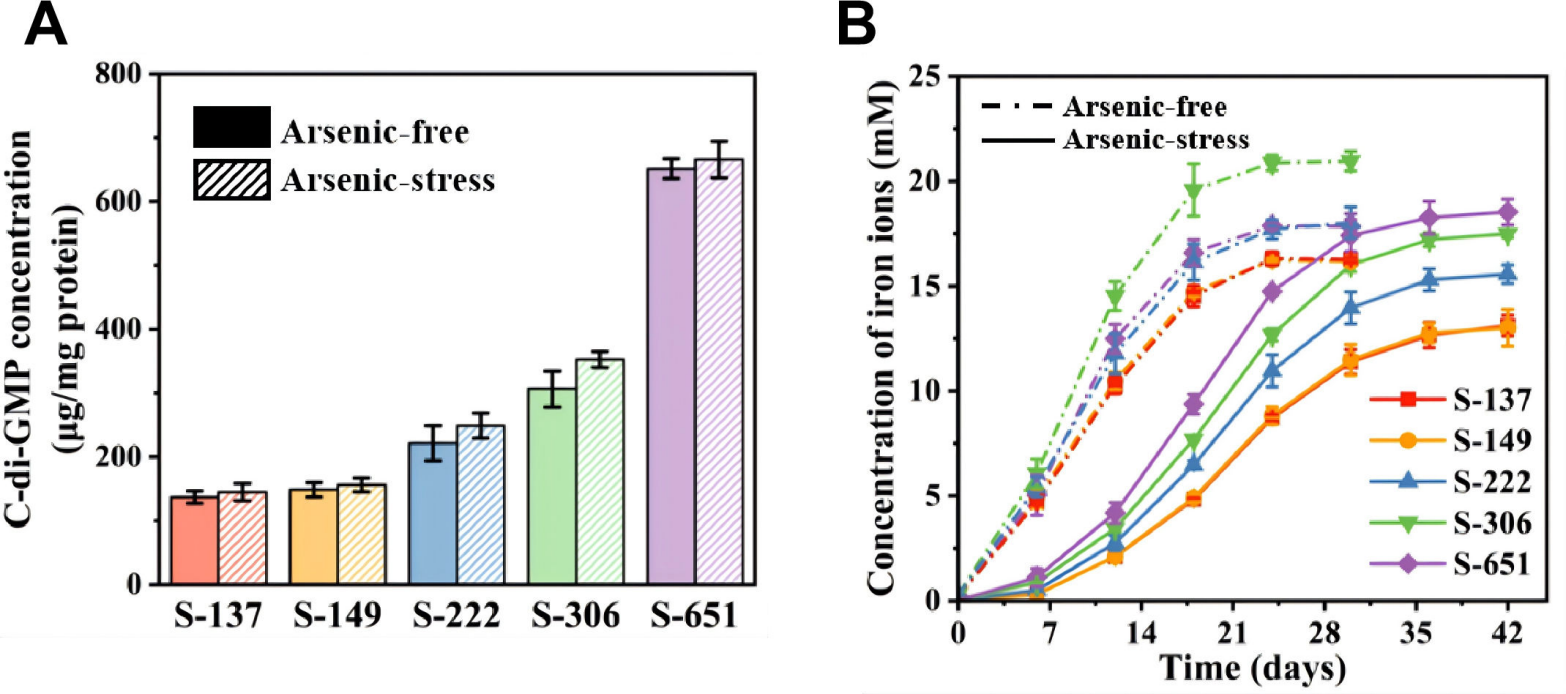
(**A**) Intracellular c-di-GMP concentrations under arsenic-free (solid columns) and arsenic-stressed (hatched columns) conditions. (**B**) Kinetic profiles of bioleaching by the strains under arsenic-free (dashed curves) and arsenic-stressed (solid curves) conditions. Data are represented as mean values ± standard deviation (SD) (*n* = 3 independent replicates).

However, these engineered strains have markedly different varying intracellular c-di-GMP levels (Fig. 2A). Strain S-149, harboring the *yedQ* gene, displayed an intracellular c-di-GMP level similar to that of the control strain S-137 with the empty plasmid vector (136.9 ± 9.3 versus 148.5 ± 11.1 μg mg^−1^ total protein). Thus, introducing the *yedQ* gene from *E. coli* into *A. ferrooxidans* fails to significantly elevate the intracellular c-di-GMP levels, potentially because the heterologously expressed YedQ protein is non-functional in the acidophilic host (33). In contrast, strains S-222, S-306, and S-651 exhibited elevated intracellular c-di-GMP levels of 221.5 ± 27.3, 306.3 ± 28.1, and 651.4 ± 15.5 μg mg^−1^ total protein, representing approximately1.7-, 2.5-, and 5-fold increases compared to the control strain (Fig. 1A). The results demonstrate that overexpressing the *A. ferrooxidans* DGC genes (*AFE_1379*, *AFE_0053*, and *AFE_1373*) effectively increases intracellular c-di-GMP levels, with the elevation order being *AFE_1373* > *AFE_0053* > *AFE_1379*.  

### Bioleaching performance of engineered strains

To investigate how variations in intracellular c-di-GMP levels affect bioleaching efficiencies of engineered strains, we systematically compared the kinetics and extents of iron release from pyrite induced by these strains under arsenic-free and arsenic-stressed conditions over 30 days (Fig. 2B). The kinetic profiles of all strains exhibited S-shaped curves, characterized by an initial lag phase for microbial attachment and biofilm development, followed by an exponential phase of rapid oxidation. The critical role of microbe-mineral interactions in iron release from pyrite was further supported by the bacteria-free control experiment, in which iron release remained negligible over the 60-day period under the conditions of this study (Fig. S3). In the absence of arsenic, the iron release rates of different strains during the exponential phases followed the order: S-306 > S-651 > S-222 > S-149 ≈ S-137 (Fig. S4A). The sulfate production rates from pyrite oxidation (Fig. S5) among the strains followed the same order. The iron release rate of strain S-306 was 1.21 ± 0.06 mM day⁻¹, which was 1.6 times that of the control strain (S-137) and significantly higher than those of S-651 (0.98 ± 0.08 mM day⁻¹) and S-222 (0.97 ± 0.04 mM day⁻¹). Strain S-149 showed a similar iron bioleaching capability to that of the control strain, consistent with their comparable intracellular c-di-GMP levels (Fig. 2A). Correspondingly, the highest extent of pyrite oxidation was observed in S-306, followed by S-651 and S-222, and lowest in the control and S-149 (Fig. S4A). However, the intracellular c-di-GMP level of S-651 was higher than that of S-306 (Fig. 2A). This apparent inconsistency under arsenic-free conditions suggests that the bioleaching capability of engineered strains does not monotonically increase with rising c-di-GMP concentrations in *A. ferrooxidans*.

In bioleaching processes, arsenic release from refractory ores, typically containing arsenic-bearing minerals (e.g., arsenopyrite and arsenian pyrite), significantly impacts microbial activity and metal extraction (42). To investigate the bioleaching capability of engineered strains under arsenic-stressed conditions, we also compared the iron bioleaching capabilities of these strains in the presence of 5 mM sodium arsenite (Fig. 2B), reflecting the concentration and speciation of arsenic commonly encountered in natural acid mine drainage (AMD) systems (43). The presence of 5 mM As(III) subtly but statistically insignificantly increased intracellular c-di-GMP concentrations in all strains, without changing their relative order (Fig. 1C). This indicates that the strain-specific arsenic tolerance in *A. ferrooxidans* stems from pre-existing differences in basal c-di-GMP levels rather than from an arsenic-induced upregulation of this second messenger. Under arsenic stress, the iron release rates ranked as S-651 > S-306 > S-222 > S-149 ≈ S-137 (Fig. S4B). The ranking of sulfate production rates among the strains (Fig. S6) mirrored that of their iron release rates. Unlike under arsenic-free conditions, S-651 became the most efficient strain, exhibiting an iron release rate of 0.61 ± 0.01 mM day⁻¹, 1.7-fold higher than that of the control strain. Although the leaching rate of S-651 under arsenic stress decreased by ~37% compared with that under arsenic-free conditions, its overall extent of iron leaching from pyrite remained nearly unchanged. In contrast, the other strains were more severely affected by arsenic stress, resulting in more pronounced decreases in both their bioleaching rates (53–56% reduction) and extents (13–20% reduction). Moreover, growth curves (Fig. 1B) showed that S-651 grew faster than the other strains under arsenic stress, whereas all strains exhibited similar growth rates in the absence of arsenic. Consequently, strain S-651, overexpressing the *AFE_1373* gene, outperforms the other engineered strains in bioleaching under arsenic stress, demonstrating superior arsenic tolerance, even though S-306 exhibits better bioleaching capability in the absence of arsenic.

To further investigate why different engineered strains exhibit varying arsenic tolerance, we compared the relative proportions and valence states of solid-associated and dissolved arsenic at the end of 42-day arsenic-stressed bioleaching experiments (Fig. S7). The dissolved arsenic was predominantly As(V), with negligible As(III), which is reasonable given that the experiments were conducted under aerobic conditions. The percentages of solid-associated arsenic, including that within mineral lattices and in surface-adsorbed forms, followed the order: S-651 (50%) > S-306 (40%) > S-222 (36%) > S-149 (30%) > S-137 (28%). This indicates that the overexpression of the endogenous DGC genes (*AFE_1373*, *AFE_0053*, and *AFE_1379*) not only enhances the bioleaching capability of *A. ferrooxidans* but also facilitates the immobilization of arsenic from aqueous environments. Specifically, S-651 with the overexpressed *AFE_137*3 gene demonstrated significantly enhanced arsenic immobilization capacity. The synchrotron XRD patterns (Fig. S8) suggest that the solid product after 42-day experiments with arsenic was scorodite (FeAsO₄·2H₂O), while jarosite (KFe₃(SO₄)₂(OH)₆) was the dominant secondary mineral after experiments under arsenic-free conditions. Thus, strain S-651, characterized by a higher growth rate (Fig. 1B) and superior bioleaching performance (Fig. S4B), can release Fe³^+^ more rapidly. This accelerates the precipitation of scorodite and reduces dissolved arsenic concentrations, which in turn mitigates arsenic toxicity and ultimately enables S-651 to exhibit more prominent bioleaching efficiency and arsenic tolerance compared to other strains. 

### Differences in biofilm characteristics of engineered strains

**Fig 3:**
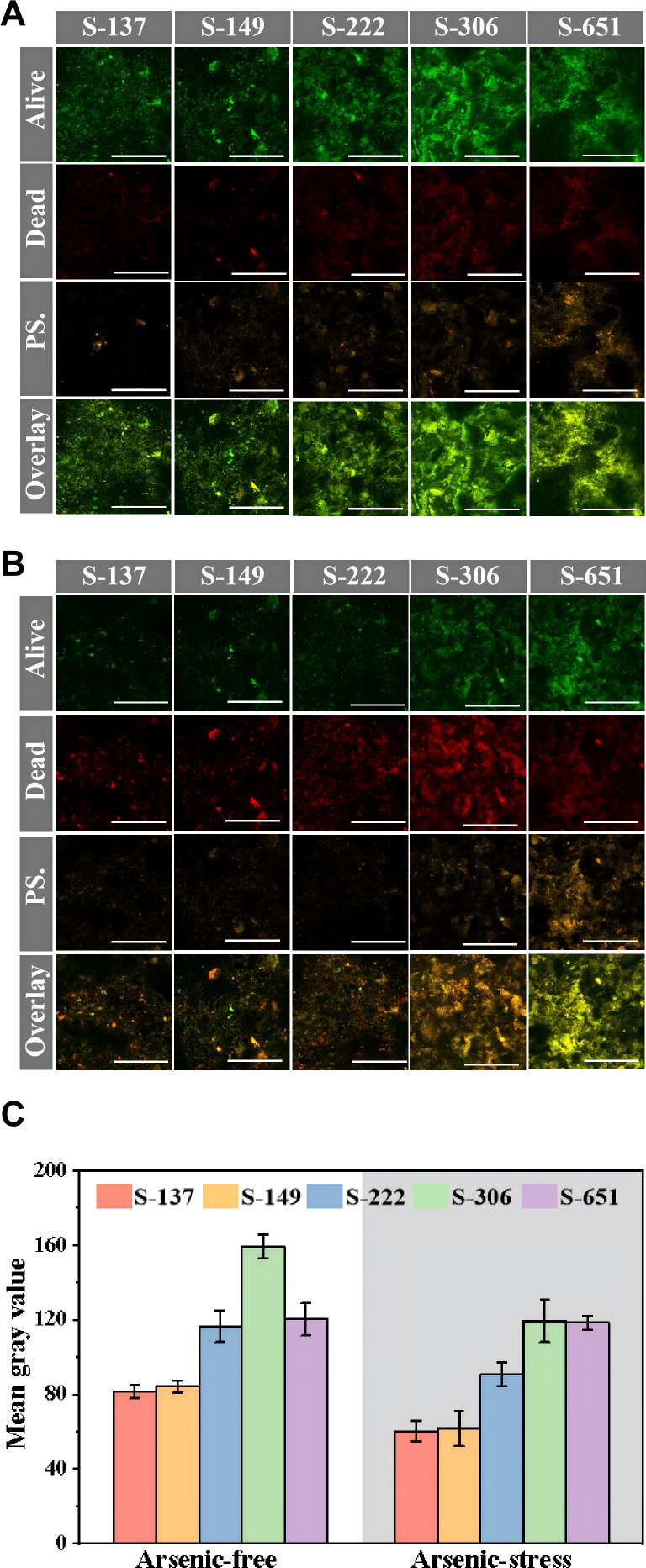
Representative CLSM images of biofilms formed by engineered *A. ferrooxidans* strains on pyrite surfaces under (**A**) arsenic-free and (**B**) arsenic-stressed conditions. The images show live cells (green), dead cells (red), exopolysaccharides (PS., orange), and merged views. Scale bar: 100 µm. (**C**) Semi-quantitative analysis of biofilm biomass via mean gray value in the merged alive and dead channels from CLSM images of the engineered strain biofilms formed on pyrite, under arsenic-free and arsenic-stressed conditions (conducted using ImageJ software, *n* = 3).

To study how varying c-di-GMP levels differentially affect biofilm properties of engineered strains under arsenic and arsenic-free conditions, cell viability, exopolysaccharides, and total cellular protein within these strains’ biofilms on pyrite-coated slides were compared. The representative CLSM images (Fig. 3A and 2B) revealed that, under arsenic-free conditions, all engineered strains maintained comparable alive/dead cell ratios, with a live cell proportion of approximately 65% (Fig. S9). In contrast, under arsenic-stressed conditions, the live cell percentages for strains S-137, S-149, S-222, and S-306 significantly decreased to 44–47%, indicating that arsenic toxicity markedly impaired microbial metabolic activity of these strains. Notably, the S-651 strain maintained a live cell percentage of 59% despite arsenic exposure, exhibiting significantly higher viability than other strains. This demonstrates S-651’s superior arsenic stress resistance, consistent with the observation that S-651 grew faster than other strains under arsenic stress (Fig. 1B).

**Fig 4:**
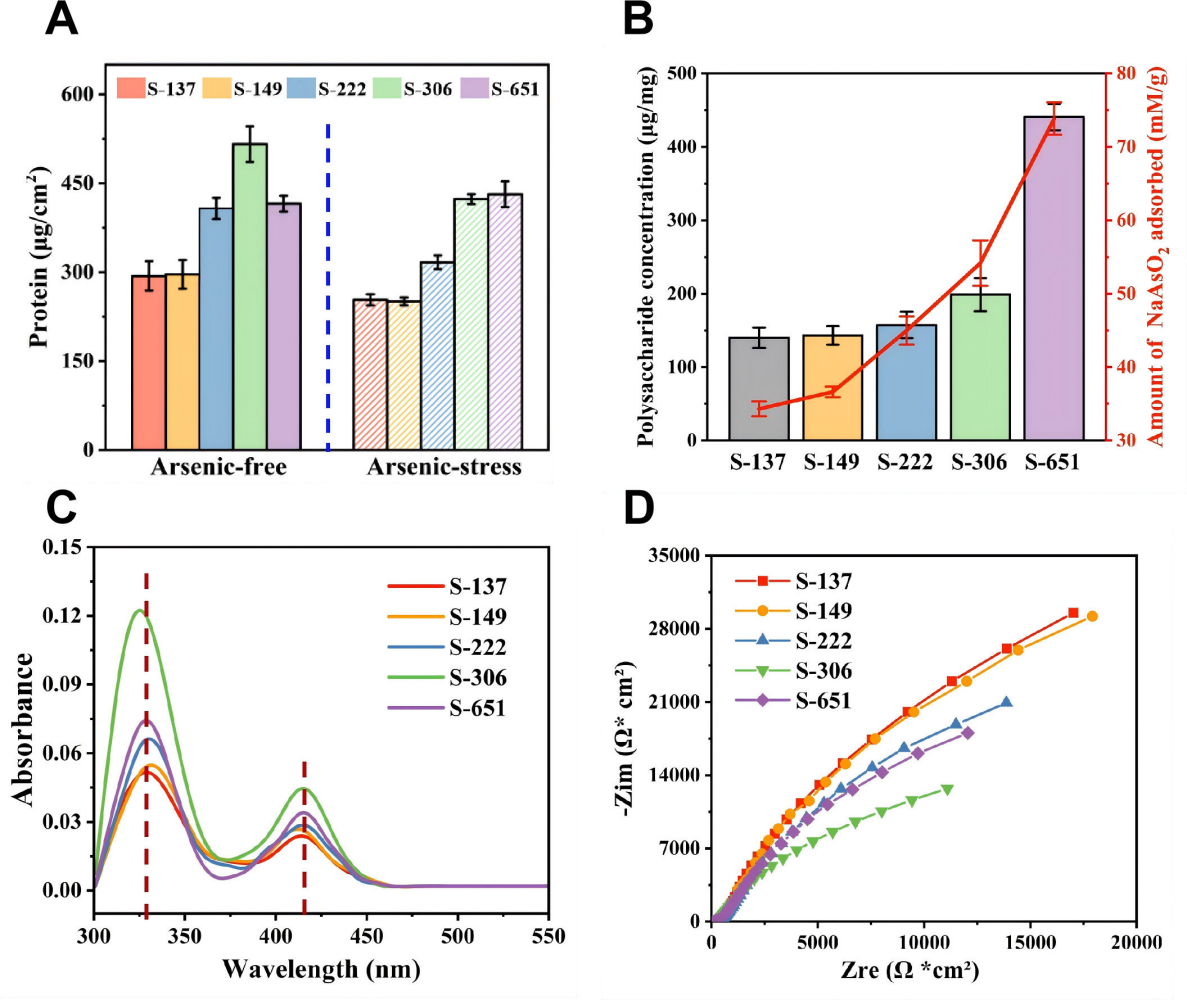
(**A**) Protein contents of biofilms formed by engineered strains on pyrite surfaces under arsenic-free (solid columns) and arsenic-stressed (hatched columns) conditions. (**B**) Polysaccharide contents (columns) in EPS and arsenite sorption capacities of EPS (red dots; with 30 mg dry weight of EPS used for sorption assays), both measured using EPS extracted from engineered strains after reaction under arsenic stress. (**C**) UV-Vis spectra of EPS from the engineered strains under arsenic stress, with dashed lines indicating characteristic peaks of *c*-type cytochromes. (**D**) Nyquist plots of the biofilms under arsenic stress, obtained via electrochemical impedance spectroscopy (EIS), depict biofilm conductivity, where a smaller semicircle radius corresponds to lower biofilm resistance and higher electron transfer efficiency. Data in panels **A** and **B** are represented as mean ± standard deviation (SD) (*n* = 3 independent replicates).

The total biomass of biofilms, estimated by densitometry analysis of live/dead merged CLSM images (Fig. 3A and 2B), followed the order: S-306 > S-651 > S-222 > S-149 ≈ S-137 under arsenic-free conditions, but shifted to S-651 > S-306 > S-222 > S-149 ≈ S-137 under arsenic-stressed conditions (Fig. 3C). The biomass of live cells within the biofilms, estimated from the live-cell CLSM images, also followed the same trend (Fig. S10). Furthermore, this pattern observed in CLSM analyses was corroborated by protein contents quantified using the bicinchoninic acid (BCA) assay (Fig. 4A). Specifically, under arsenic-free conditions, the total protein content of the S-306 biofilm was 515 ± 30 µg cm⁻², which was notably higher than that of the control strain S-137 (296 ± 25 µg cm⁻²). The presence of arsenic reduced the total protein contents of biofilms in all strains to varying degrees, except for S-651. Strikingly, S-651 sustained robust biofilm development, with protein contents of 420 ± 13 µg cm⁻² without arsenic and 425 ± 21 µg cm⁻² with arsenic, showing remarkable resilience and no significant arsenic-induced biomass loss. The results suggest that the overexpression of the endogenous DGC genes (*AFE_1373*, *AFE_0053*, and *AFE_1379*) could increase the total biomass and thickness of *A. ferrooxidans* biofilms on pyrite to varying degrees. Specifically, S-306 with the overexpressed *AFE_0053* gene formed markedly thickest biofilms in the absence of arsenic, whereas under arsenic stress, strain S-651 with the overexpressed *AFE_1373* gene exhibited a stronger ability to develop biofilms on pyrite surfaces (Fig. 3 and 4A).

Additionally, the representative CLSM images using ConA-TRITC labeling (Fig. 3) revealed that the fluorescence intensity of exopolysaccharides increased with the overexpression of the endogenous DGC genes (*AFE_1373, AFE_0053*, and *AFE_1379*), regardless of arsenic exposure. Under arsenic-free conditions, strain S-306 formed a thicker biofilm with greater biomass, but its exopolysaccharide content was still lower than that of strain S-651. Thus, the biofilm of S-306 had a higher protein proportion, while that of S-651 was characterized by a greater exopolysaccharide proportion. Under arsenic-stressed conditions, the exopolysaccharide fluorescence of S-651 exceeded that of S-306 even further. This trend was confirmed by the polysaccharide contents determined using the Phenol-Sulfuric Acid Assay Kit (Fig. 4B). For example, the exopolysaccharide content of S-651 (441 ± 18 µg/mg) was 3.2-fold and 2.2-fold higher than those of the control strain S-137 (140 ± 14 µg/mg) and S-306 (199 ± 23 µg/mg) under arsenic-stressed conditions. The relatively high exopolysaccharide abundance in the S-651 biofilm likely contributed to its superior arsenic stress resistance (Fig. 1B), as exopolysaccharides typically serve as critical physicochemical barriers for immobilizing heavy metals (16). Consistently, the FTIR spectrum of S-651’s EPS (Fig. S11) showed stronger peaks at 1080, 3300, and 1630 cm⁻¹, corresponding to polysaccharide bonds, hydroxyl, and carboxyl/amide groups, respectively, indicative of abundant arsenic-binding functional groups (44). In line with this, arsenic adsorption experiments (Fig. 4B) using equal-mass EPS from different engineered strains demonstrated that higher exopolysaccharide proportions in EPS correlated with significantly greater arsenic adsorption capacity. For instance, the EPS of S-651 exhibited 2.2-fold and 1.4-fold higher equilibrium arsenic adsorption capacities than those of the control strain S-137 and strain S-306, respectively. Thus, overexpression of the gene *AFE_1373* not only markedly elevated the intracellular c-di-GMP level in S-651 but also increased the relative proportion of exopolysaccharides and functional groups in its biofilm, thereby strengthening biofilm barrier function, promoting arsenic sequestration, mitigating cellular toxicity, and ultimately improving bioleaching resilience.

**Fig 5:**
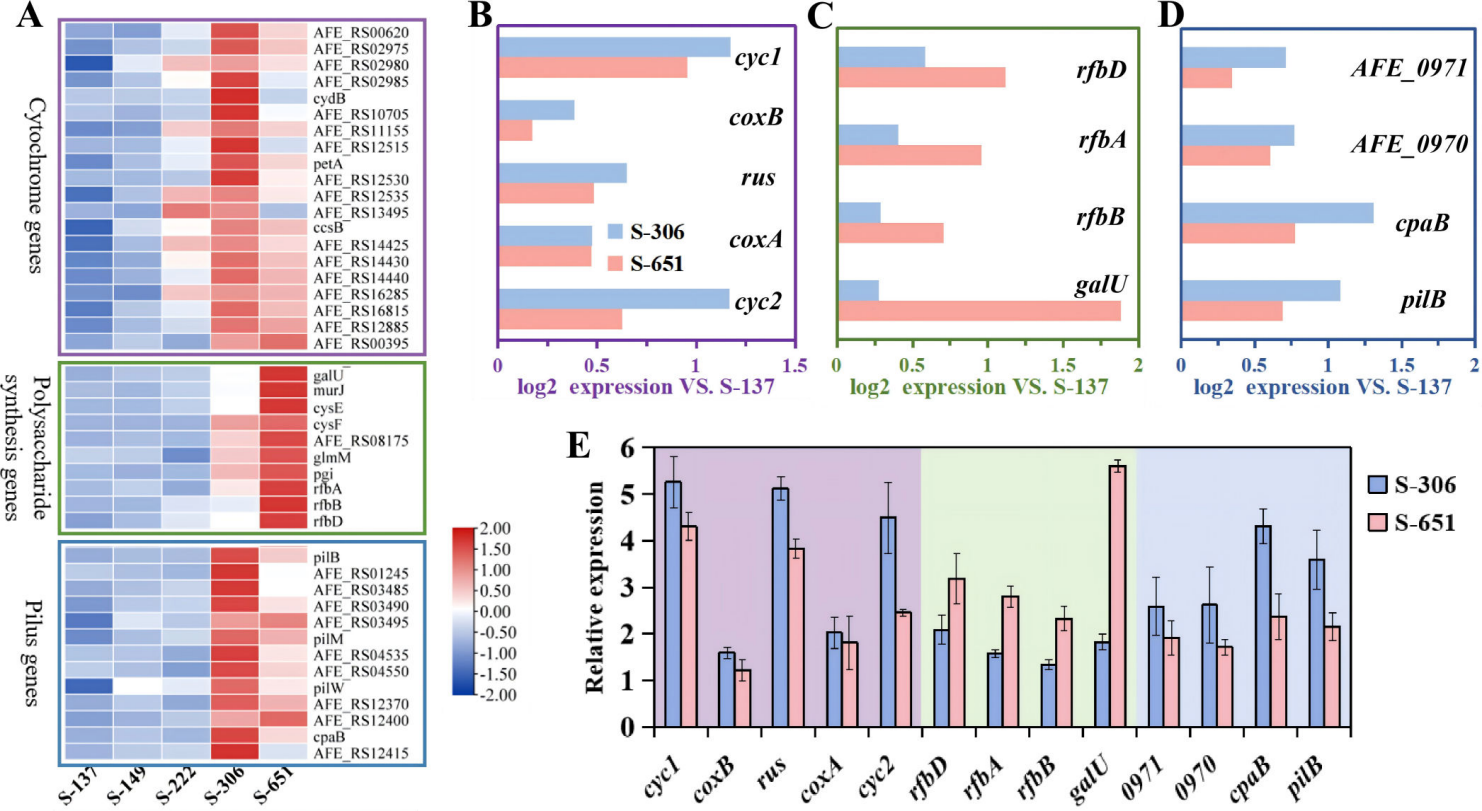
Transcriptomic analysis of *A. ferrooxidans* biofilm cells under arsenic stress. (**A**) Heatmap of DEGs related to (*c*-Cyts), polysaccharides, and pilus in strains S-137 (control), S-149, S-222, S-306, and S-651. Comparative expression profiles of genes encoding (**B**) key cytochromes associated with extracellular electron transfer (EET), (**C**) exopolysaccharide biosynthesis, and (**D**) pilus biosynthesis in strains S-306 (blue) and S-651 (red) relative to S-137. (**E**) Quantitative PCR (qPCR) validation of DEGs in S-306 (blue) and S-651 (red): *c*-Cyt genes (purple background), exopolysaccharide biosynthesis genes (green background), and pilus biosynthesis genes (blue background). Data in panel **E** are represented as mean ± standard deviation (SD) (*n* = 3 independent replicates).

In comparison, the overexpression of the gene *AFE_0053* increased the protein proportion in the S-306 biofilm relative to that of S-651, as confirmed by the UV-vis spectra of biofilm lysates from the two strains (Fig. 4C). Specifically, the absorbances at 320–330 and 410–420 nm, characteristic of *c*-type cytochromes (*c*-Cyts) (45), were higher in S-306 than in S-651 and other stains. *c*-Cyts on the outer cell membrane of *A. ferrooxidans* play a crucial role in transferring electrons from extracellular pyrite to cytochrome oxidase in the plasma membrane (46). Thus, the enhanced proportion of *c*-Cyts in the S-306 biofilm likely facilitated extracellular electron uptake and promoted pyrite oxidation. This was supported by electrochemical impedance spectroscopy (EIS) (Fig. 5D), which showed a markedly lower internal resistance in S-306 biofilms. The corresponding Nyquist plots revealed a significantly smaller arc radius for S-306 than the other strains, indicating lower resistance and favorable electron transfer in its biofilm, consistent with the elevated *c*-Cyt content (Fig. 4D). These results indicate that differences in the relative proportions of exopolysaccharides and cytochrome proteins in the biofilms of S-651 and S-306 likely contribute to their distinct capabilities in bioleaching and arsenic stress resistance.

### Gene expression profiling of biofilm cells

To further elucidate how DGC gene regulation influences biofilm development, EPS matrix compositions, bioleaching performance, and arsenic stress resistance in *A. ferrooxidans*, we compared the gene expression profiles of engineered strains (S-149, S-222, S-306, and S-651) to that of the control strain S-137 via transcriptomic analysis. Total RNA was extracted from the biofilm cells at the iron release peaks during bioleaching experiments for subsequent transcriptome profiling. A total of 2492 genes of *A. ferrooxidans* were detected, and the heatmap of all these genes is illustrated in Fig. S12A. Principal component analysis (PCA) of the transcriptomes further revealed distinct physiological states among the biofilm cells of the different strains (Fig. S12B). Differential expression analysis relative to the S-137 control, using negative binomial tests (*P* < 0.05, fold-change ≥ 2), identified strain-specific sets of differentially expressed genes (DEGs): S-149 (9 DEGs: 1 upregulated, 8 downregulated), S-222 (11 DEGs: 10 upregulated, 1 downregulated), S-306 (57 DEGs: 52 upregulated, 7 downregulated), and S-651 (237 DEGs: 223 upregulated, 14 downregulated) (Fig. S12). Collectively, these results indicate that DGC genes primarily regulate *A. ferrooxidans* through transcriptional upregulation of the majority of functional genes, with the extent of transcriptional reprogramming correlating with the level of c-di-GMP modulation. InterProScan-based domain analysis revealed no annotated non-GGDEF domains in AFE_1379 or AFE_0053, whereas AFE_1373 possesses additional P-loop NTPase and IstB_IS21-related domains. These auxiliary domains may provide extra regulatory inputs and could partly contribute to the distinct expression profile observed in strain S-651, in addition to the elevated intracellular c-di-GMP level.

Bioleaching experiments (Fig. 2B) revealed that S-306 demonstrated the highest bioleaching capability under arsenic-free conditions, whereas S-651 exhibited superior arsenic resistance and maintained optimal bioleaching performance under arsenic stress. We therefore focused on DEGs with divergent expression patterns between S-306 and S-651 to elucidate the mechanistic basis for their respective phenotypic advantages. As shown in Fig. 5A, S-306 exhibited the highest expression of key *c*-Cyts genes (*cyc1*, *coxB*, *rus*, *coxA*, and *cyc2*) among all engineered strains. S-651 also showed elevated expression relative to the control, but levels were lower than in S-306 (Fig. 5B). This trend was confirmed by quantitative PCR (qPCR) (Fig. 5E), showing that the expression of these genes in S-306 was 1.1 to 1.8-fold higher than in S-651. The higher expression of these *c*-Cyts genes in S-306 is consistent with the stronger peaks assigned to *c*-Cyts in the UV-vis spectrum of its biofilm lysates (Fig. 4C) and with the lower biofilm resistance revealed by EIS (Fig. 4D). Overall, the overexpression of the DGC gene *AFE_0053* significantly upregulates the expression of *c*-Cyts genes and enhances electron transfer from pyrite to *A. ferrooxidans*, indicating that this gene expression does not increase monotonically with c-di-GMP levels.

Transcriptome data also showed that exopolysaccharide biosynthesis genes (*rfbD*, *rfbA*, *rfbB,* and *galU*) were upregulated in both S-306 and S-651 relative to the control, with S-651 exhibiting the highest expression (Fig. 5A and C). Their expression generally increased with intracellular c-di-GMP concentrations, consistent with previous reports that elevated c-di-GMP promotes exopolysaccharide production and biofilm formation, thereby enhancing stress resistance (47, 48). This trend was confirmed by the CLSM images (Fig. 3), and qPCR further validated that the expression in S-651 was 1.5–3.1-fold higher than that in strain S-306 (Fig. 5E). Moreover, genes encoding arsenic resistance were not differentially expressed among all strains after arsenic exposure (Fig. S13), indicating that the superior arsenic tolerance of S-651 with DGC gene of *AFE_1373* was not due to upregulation of arsenic detoxification genes but likely arose from enhanced exopolysaccharide production, which acts as a barrier limiting arsenic entry and toxicity.

Notably, pilus biosynthesis genes (*AFE_0091*, *AFE_0090*, *cpaB*, and *pilB*) were significantly upregulated in both S-306 and S-651 compared to the control strain S-137, with a more pronounced increase in S-306 (Fig. 5A and D). Consistently, qPCR results confirmed that the expression of these genes in S-306 was 1.4–1.8-fold higher than in S-651 (Fig. 5E). Since *A. ferrooxidans* colonizes solid surfaces through pili-mediated sliding, twitching motility, and adhesion (49), this upregulation likely facilitated its attachment to pyrite surface and subsequent biofilm formation. The stronger induction of pilus biosynthesis genes in S-306 compared to S-651 likely contributes to its ability to develop thicker biofilms on pyrite under arsenic-free conditions (Fig. 3A).

### Regulatory mechanisms of c-di-GMP on biofilm matrix, bioleaching performance, and arsenic resistance in *A. ferrooxidans*

Our results demonstrate that overexpressing the endogenous DGC genes *AFE_1373*, *AFE_0053*, and *AFE_1379* into *A. ferrooxidans* produced engineered strains S-222, S-306, and S-651, respectively. These strains exhibited similar growth kinetics under arsenic-free conditions but distinct, progressively elevated intracellular c-di-GMP levels (S-222 < S-306 < S-651). Compared with the control strain S-137, carrying the empty pYDT plasmid, the engineered strains generally formed thicker biofilms and exhibited higher bioleaching capabilities. However, the rates and extents of pyrite oxidation did not increase monotonically with elevated c-di-GMP levels. Under arsenic-free conditions, S-306, with the overexpressed *AFE_0053* gene and a moderate intracellular c-di-GMP concentration (306.3 ± 28.1 μg mg^−1^ protein), exhibited optimal biofilm formation and pyrite oxidation capability (Fig. 1 to 3). Its biofilms were enriched in *c*-Cyts (Fig. 4 and 5), which facilitates extracellular electron transfer from pyrite to intracellular electron transport carriers and reduces internal resistance of the biofilm. As an obligate autotroph, *A. ferrooxidans* relies on energy-intensive carbon fixation via the Calvin-Benson-Bassham (CBB) cycle (50, 51). The increased c-di-GMP levels in S-306 enhanced the *c*-Cyts content and electrical conductivity of its biofilm, which in turn promotes electron uptake from pyrite and energy supply to the CBB cycle. Additionally, upregulation of pilus genes induced by the overexpression of the *AFE_0053* gene (Fig. 5C and E) further facilitates bacterial colonization on pyrite surfaces, leading to the formation of thicker biofilms (Fig. 3) and the higher pyrite oxidation rates (Fig. 2B). The mechanism by which intracellular c-di-GMP promotes the synthesis of *c*-Cyts and pili in *A. ferrooxidans* remains unclear, likely due to its regulation through diverse effector proteins with distinct roles (52). Nevertheless, similar phenomena have been reported in other systems, where increased c-di-GMP levels stimulate the synthesis of c-Cyts involved in EET pathways of dissimilatory iron-reducing bacteria (*Shewanella* spp. and *Geobacter* spp.), thereby enhancing the electron transfer efficiency of biofilms (33, 53, 54). Likewise, c-di-GMP has been shown to regulate the assembly and function of type IV pili in diverse bacteria, such as *Myxococcus xanthus*, *Pseudomonas aeruginosa*, and *Clostridium difficile* (48).

In contrast, S-651, which harbors the highest intracellular c-di-GMP concentration, forms thinner biofilms and exhibits relatively lower bioleaching performance than S-306 under arsenic-free conditions. Overexpression of the *AFE_1373* gene increased the intracellular c-di-GMP concentration to 651.4 ± 15.5 μg mg^−1^ total protein, resulting in the upregulation of polysaccharide synthesis genes (Fig. 5C) and enhanced production of extracellular polysaccharides within the biofilm matrix (Fig. 3). Although these polysaccharides promote bacterial attachment to pyrite surfaces and biofilm formation, they are non-conductive. The higher proportion of polysaccharides increases the internal resistance of biofilms (Fig. 4D), hindering electron uptake from pyrite to intracellular redox-active components of S-651 and thereby reducing its bioleaching performance and biofilm growth relative to S-306, which forms cytochrome-enriched biofilms.

However, in arsenic-contaminated systems, S-651 biofilms contained abundant exopolysaccharides, which served as an effective barrier against arsenic stress. These exopolysaccharides immobilized arsenic via adsorption (∼50% sequestration) and promoted scorodite precipitation, thereby reducing cellular arsenic toxicity and maintaining growth rates comparable to those under arsenic-free conditions (Fig. 1B). Although its bioleaching rate was moderately reduced to 0.61 ± 0.01 mM/day (a 37% decrease) compared to arsenic-free conditions, S-651 exhibited markedly superior arsenic resistance relative to other strains and achieved a similar extent of pyrite oxidation as in the absence of arsenic. In contrast, arsenic exposure caused a sharp decline in the proportion of viable cells in S-306 (Fig. S9), which severely impaired its metabolic activity and diminished its pyrite oxidation capability. These findings highlight a potential strategy to enhance *A. ferrooxidans* tolerance to heavy metal stress by elevating the relative abundance of extracellular exopolysaccharides in biofilms through c-di-GMP modulation.

Bacteria regulate intracellular c-di-GMP levels through the coordinated activities of DGCs and PDEs. Environmental cues, such as nutrient availability, oxidative stress, and host factors, regulate the expression or activity of DGCs and PDEs, thereby modulating c-di-GMP concentrations across critical thresholds. This dynamic control allows c-di-GMP to interact with effectors that possess different affinities, ultimately influencing physiological behaviors such as motility, attachment, and aggregation (55). However, this study shows that although both S-306 and S-651 exhibit elevated intracellular c-di-GMP levels and form thicker biofilms on pyrite compared with the control strain (S-137), the relative proportions of their biofilm components differ markedly. It is thus likely that, in both strains, c-di-GMP concentrations exceed the threshold required for the transition from motility to attachment and biofilm formation. Nevertheless, S-306 preferentially enhances the production of *c*-Cyts and pili, promoting pyrite oxidation and biofilm development, whereas S-651 favors exopolysaccharide production, leading to thicker biofilms with a greater capacity to sequester heavy metals. These results suggest that *A. ferrooxidans* may finely regulate intracellular c-di-GMP levels, even above the threshold required for biofilm formation, to adjust the relative proportions of polysaccharides and proteins within biofilms, thereby potentially enabling adaptation to varying environmental demands for iron oxidation and heavy metal resistance. However, the molecular mechanisms by which *A. ferrooxidans* precisely adjusts biofilm matrix composition through regulation of c-di-GMP levels remain to be elucidated and warrant further investigation.

### Conclusion

**Fig 6:**
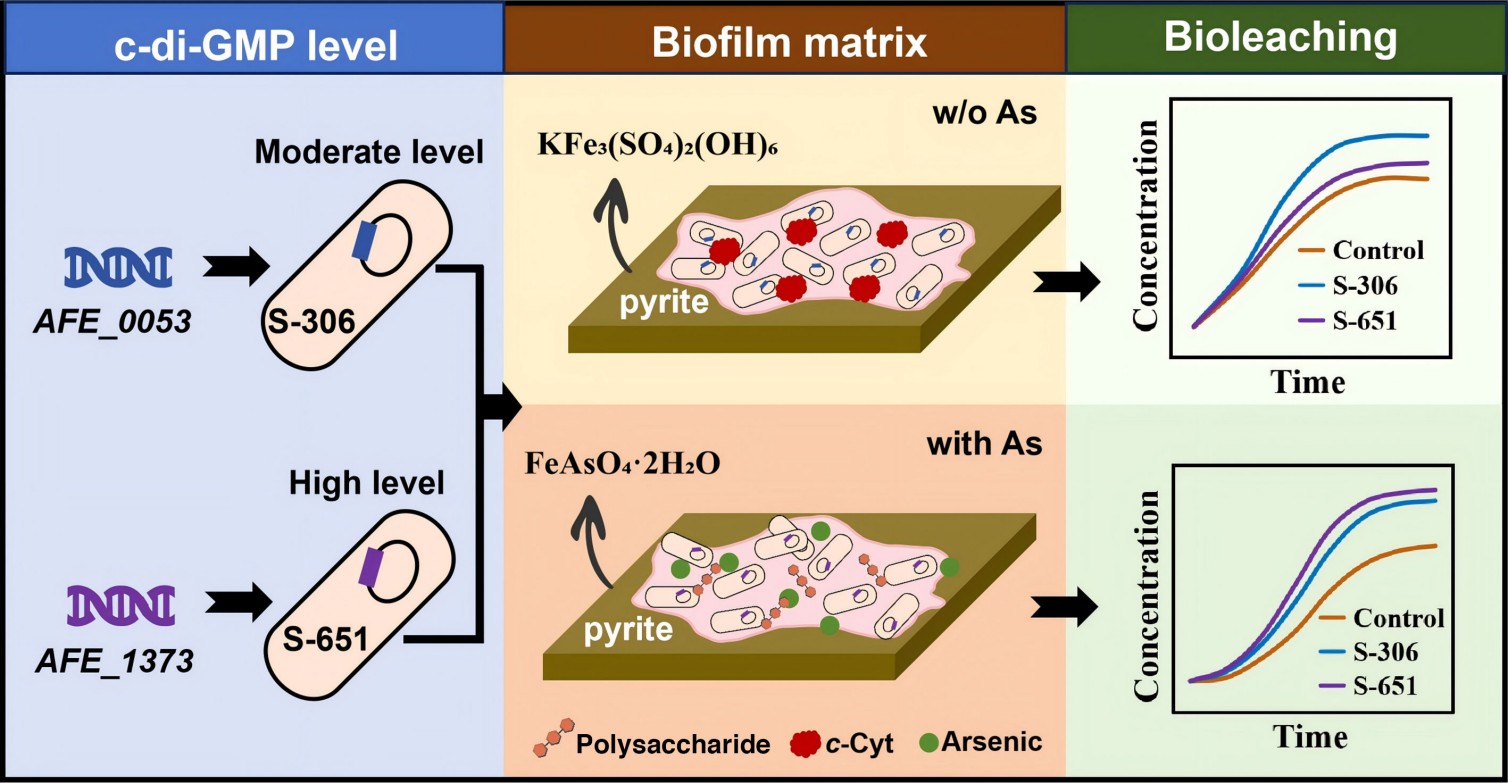
c-di-GMP–mediated modulation of biofilm matrix composition and pyrite bioleaching in engineered *A. ferrooxidans*. Strain S-306, engineered for moderate intracellular c-di-GMP levels (via *AFE_0053* expression), forms cytochrome-enriched biofilms that enhance pyrite oxidation under arsenic-free conditions. In contrast, strain S-651, engineered for high intracellular c-di-GMP levels (via *AFE_1373* expression), develops polysaccharide-rich biofilms that immobilize dissolved arsenic, providing enhanced arsenic resistance during bioleaching.

The findings of this study reveal that by modulating intracellular c-di-GMP levels through the introduction of different DGC genes, *A. ferrooxidans* can adjust the relative proportions of biofilm polysaccharides and proteins, enabling enhanced biofilm development, improved bioleaching performance, and increased tolerance to heavy metals such as arsenic (Fig. 6). This adaptive strategy probably enables *A. ferrooxidans* to persist as a dominant species under the fluctuating stressors of mining environments, such as variable heavy metal concentrations, intermittent nutrients, and sulfide oxidation-induced oxidative stress, and drive iron/sulfur cycling that shapes metal (im)mobilization. Practically, cytochrome-enriched biofilms, as in strain S-306, enhance extracellular electron transfer, improving bioleaching efficiency for metal recovery, particularly from low-grade sulfidic ores or tailings. In contrast, polysaccharide-rich biofilms, as observed in strain S-651, can effectively sequester dissolved metals, highlighting potential applications in the bioremediation of contaminated mine sites or industrial effluents. These insights further guide the development of scenario-tailored engineered strains: under heavy metal stress (a primary limiter of bioleaching performance via microbial growth inhibition), the focus should be on modifying genes for polysaccharide synthesis and heavy metal detoxification. In metal-free environments, bioleaching can be enhanced by overexpressing genes for EET-related c-Cyts and pilus biosynthesis. In future work, a single DGC placed under an inducible promoter could be used to titrate intracellular c-di-GMP and quantitatively relate c-di-GMP levels to biofilm matrix traits and bioleaching performance. Beyond bioleaching, these mechanisms can be further extended to other heavy metal-laden industrial applications mediated by *A. ferrooxidans*, such as recovery of precious metals from electronic waste, biorefining of spent batteries, and bioremediation of contaminated sites. Collectively, this work bridges microbial physiological regulation, environmental adaptation, and applied biotechnologies, providing a framework for advancing sustainable metal recovery and environmental remediation in mining and broader industrial contexts.

## Data Availability

The transcriptome data generated in this study have been deposited in the NCBI Gene Expression Omnibus (GEO) database under the accession number GSE294636. The authors declare that all data supporting the findings of this study are available within the article and its supplemental material or from the corresponding author upon reasonable request.

## SUPPLEMENTAL MATERIAL

Supplemental material aem.02288-25-s0001.docx Supplemental methods, Fig. S1 to S14, and Tables S1 to S3.

ASM does not own the copyrights to Supplemental Material that may be linked to, or accessed through, an article. The authors have granted ASM a non-exclusive, world-wide license to publish the Supplemental Material files. Please contact the corresponding author directly for reuse.
