# Supplementary material for: Biofilm engineering through c-di-GMP tuning boosts bioleaching efficiency and arsenic tolerance in Acidithiobacillus ferrooxidans
Source: Appl Environ Microbiol. 2026 Feb 18;92(3):e02288-25. doi: 10.1128/aem.02288-25 (PMC12997845; doi:10.1128/aem.02288-25)
Supplement: Supplemental material — Supplemental methods, Fig. S1 to S14, and Tables S1 to S3. [file aem.02288-25-s0001.docx]

Supplementary Information

**Biofilm Engineering Through c-di-GMP Tuning Boosts Bioleaching Efficiency and Arsenic Tolerance in *Acidithiobacillus ferrooxidans***

Xi Han^1^, Yidan Hu^2,^#, Yanbo Yue^2^, Yuefei Ding^1^, Bin Cao^3^, Liang Shi^2,4^, and Juan Liu^1,^#

^1^ The Key Laboratory of Water and Sediment Sciences, College of Environmental Sciences and Engineering, Peking University, Beijing, 100871, China.

^2^ Department of Biological Sciences and Technology, School of Environmental Studies, China University of Geosciences, Wuhan, 430074, China.

^3^ Gene and Linda Voiland School of Chemical Engineering and Bioengineering, Washington State University, Pullman, 99163, Washington, United States.

^4^ State Key Laboratory of Geomicrobiology and Environmental Changes, China University of Geosciences, Wuhan 430074, China.

#**Corresponding Authors**:

Email addresses: [juan.liu@pku.edu.cn](mailto:juan.liu@pku.edu.cn) (J Liu), huyidan@cug.edu.cn (Y Hu)

| 24 Pages |
| --- |
| 4 Sections ………. Page S1-S6 |
| 14 Figures ……… Page S7-S20 |
| 3 Tables ……… Page S21-S23  References ………… Page S24 |

**Section S1. Construction of plasmid pYDT**

The pYYDT plasmid, a derivative of the pBBR vector series, is a Biobrick-compatible vactor that facilitates multiple gene assembly. pBBR-based plasmids can be replicated in *A. ferrooxidans*. Moreover, the retention of the mob gene from the broad-host-range plasmid pBBR1-MCS2 within pYYDT enables efficient conjugative transfer. However, the shared kanamycin resistance marker (KmR) between pYYDT and the donor *Escherichia coli* SM10 strain complicated the selection of transformants. To resolve this, we replaced the kanamycin resistance cassette with a streptomycin resistance gene (SmR) via homologous recombination. The resulting modified plasmid was designated pYDT. The stability of the pYDT vector in *A. ferrooxidans* was improved through a plasmid-host adaptation strategy (1). The copy number of the pYDT vector was determined using previously reported methodologies (2). In brief, the detection of both plasmid and chromosomal DNA from *A. ferrooxidans* was performed via quantitative polymerase chain reaction (qPCR) using two sets of primers specific for the *lacI* gene on the plasmid and for *alaS* on the chromosomal DNA, respectively. Given that both *lacI* and *alaS* are single-copy genes in pYDT and chromosomal DNA, the plasmid copy number can be calculated as the ratio of *lacI* copies to *alaS* copies. The copy number of the pYDT vector was found to be 52 ± 6 and 45 ± 1 (Fig. S14) before and after the bioleaching experiment lasting 45 days without antibiotics, respectively, indicating the relative stability of pYDT vector in *A. ferrooxidans*.

**Section S2.** **Quantification of ferrous ions, sulfate ions, and polysaccharides.**

Concentrations of ferrous ions were quantified using the ferrozine colorimetric method, following previous published procedures (3). Samples were collected at defined intervals using sterile syringes, immediately filtered through 0.22 μm polyethersulfone membranes (Millipore), and preserved in pre-acidified vials containing 0.5 M HCl to stabilize valence state. Dilutions were performed with 0.5 M HCl to ensure concentrations fell within the 1–100 μM calibration range. The ferrozine solution contained: 0.2 g/L ferrozine, 12 g/L HEPES, and 10 g/L hydroxylamine hydrochloride. For analysis, 38 μL of the sample was combined with 1.5 mL of the ferrozine solution in 1.5 mL tube, thoroughly mixed, and incubated for 10 minutes. Absorbance was measured at 562 nm against reagent blanks using a UV-2600i spectrophotometer (Shimadzu, Japan) with matched quartz cuvettes (1 cm pathlength). Iron ion concentrations were calculated from a daily-generated 5-point calibration curve (R² ≥ 0.99) prepared from certified FeSO₄·7H₂O standards (NIST-traceable, AccuStandard). Quality control included triplicate measurements and analysis of matrix blanks.

Sulfate concentrations were determined by ion chromatography (ICS-1100, Thermo Fisher Scientific, USA) (4). The mobile phase consisted of 4.5 mM Na₂CO₃ / 1.4 mM NaHCO₃ prepared in ultrapure water (18.2 MΩ·cm; Milli-Q), with an isocratic flow rate of 1.2 mL/min. Sample injections of 25 μL were performed using an AS-DV autosampler after 0.22 μm polyethersulfone membranes (Millipore) and appropriate dilution with ultrapure water to maintain concentrations within the 1–100 mg/L linear calibration range (R² ≥ 0.99). Quantification was performed by peak area comparison against potassium sulfate (K₂SO₄; certified reference material, Sigma-Aldrich) calibration standards.

**Section S3. Preparation of pyrite-coated glass slides and characterization of biofilms.**

Mineral-coated glass slides were prepared according to the protocol established previously (5). Briefly, sterile glass slides (area: 1.44 cm²) were first coated with a mixture of 7.5 g/L gelatin and 0.75 g/L potassium dichromate and then air-dried. Subsequently, 0.1 g of pretreated pyrite was resuspended in 100 μL of ultrapure water and evenly coated onto the prepared slides. Coated slides underwent overnight desiccation in an anaerobic chamber followed by UV sterilization (30 min per side) prior to transfer to 12-well plates containing 2 mL 9K medium (pH 2 ± 0.2, Table S3). *A. ferrooxidans* strains cultured in 9K medium (Table S3) supplemented with 1% (w/v) elemental sulfur for 7 days, then harvested, washed twice in sulfur-free 9K medium, and adjusted to OD₆₀₀ = 0.10 ± 0.01, were inoculated in the 12-well plates. The cultures were incubated at 30°C without shaking. The *A. ferrooxidans* cells formed biofilms on pyrite-coated glass slides.

### Biofilms formed on pyrite-coated glass slides were analyzed in terms of (i) total protein content, (ii) cell viability, (iii) exopolysaccharide content, and (iv) cytochrome content. (i) For total protein content quantification, slides collected at specific time points were incubated in 2 mL of 0.2 M NaOH at 98 ℃ for 2 h. Supernatants (50 μL) were mixed with acetone (200 μL), incubated at 4 ℃ for 1 hour, centrifuged at 20,000 ×g for 30 minutes. Then, the pellet was resuspended in 50 μL ultrapure water. Biomass was determined using a BCA protein assay kit (Thermo Fisher Scientific, USA). (ii) For cell viability, harvested slides were stained with the Live/Dead BacLight Bacterial Viability Kit (#L13152, Thermo Fisher Scientific, USA) for 20 minutes, rinsed twice with PBS. (iii) For exopolysaccharide contents, slides were stained with ConA-TRITC (#C860, Thermo Fisher) for 30 min to label exopolysaccharides, followed by PBS rinses. Stained slides were observed using a confocal laser scanning microscope (CLSM, TCS SP8, Leica, Germany), with optimized excitation/emission settings for SYTO 9 (488/550 nm), PI (535/617 nm), and ConA-TRITC (557/576 nm). The polysaccharide content within biofilms was quantified using a Phenol-Sulfuric Acid Assay Kit (Cat. No. ADS-W-TDX061-48, Jiangsu Aidisheng Biological Technology Co., Ltd., China). Briefly, 10 mg of lyophilized EPS powder was dissolved in 10 mL distilled water by heating at 95°C in a water bath until complete dissolution to prepare the polysaccharide test solution. Aliquots (100 μL) of this solution were transferred to 1.5 mL microcentrifuge tubes, followed by sequential addition of 50 μL Reagent One (phenol solution) and 250 μL concentrated sulfuric acid. After immediate vortex mixing, samples were incubated at 95°C for 30 min and subsequently cooled to room temperature for 15 min prior to analysis. Absorbance measurements were performed at 488 nm using a UV-Vis spectrophotometer (UV-2600i, Shimadzu, Japan). Polysaccharide concentrations were determined against a five-point calibration curve (R²≥ 0.99) generated with the kit-provided glucose standard, with all samples analyzed in technical triplicates. (iv) For cytochrome analysis, biofilms grown on the slides for 24 days were harvested, disrupted by ultrasonication on ice (5 min, JY92-IIN, Scientz, China), and centrifuged (12,000 rpm, 5 min). The supernatant was analyzed using UV-vis spectrophotometry (UV-2600i, Shimadzu, Japan) across 300 -700 nm to quantify cytochrome content.

**Section S4. EPS extraction and arsenic adsorption assays.**

To investigate the effects of EPS compositions in *A. ferrooxidans* biofilms on arsenic immobilization, EPS were extracted from stationary-phase bacterial cultures using an ethanol precipitation method (6). Cultures were centrifuged at 8,000 rpm and 4 ℃ for 10 minutes to collect the supernatant. This was mixed with three volumes of ice-cold ethanol, incubated at 4 ℃ for 24 hours, and centrifuged to collect EPS precipitates. The precipitates were resuspended in ultrapure water, dialyzed using 12 kDa cellulose membranes against ultrapure water for 24 hours, and lyophilized using freeze-dryer (FD-1A-50, Biocool, China). The resulting EPS powder was stored at -20 °C. For the arsenic adsorption experiments by EPS, 30 mg of lyophilized EPS powder was incubated in 10 mL of 9K medium with 5 mM NaAsO₂ at 30 °C for 24 hours, and solutions were collected before and after incubation, filtered through 0.22 μm filters, and analyzed for arsenic speciation and concentration using high-performance liquid chromatography coupled with inductively coupled plasma mass spectrometry (HPLC-ICP-MS) (1260 HPLC + 7850 ICP-MS, Agilent, USA). Total run time for each sample was 4.5 min, with arsenate and arsenite eluting at retention times of 1.9 minutes and 2.5 minutes, respectively.


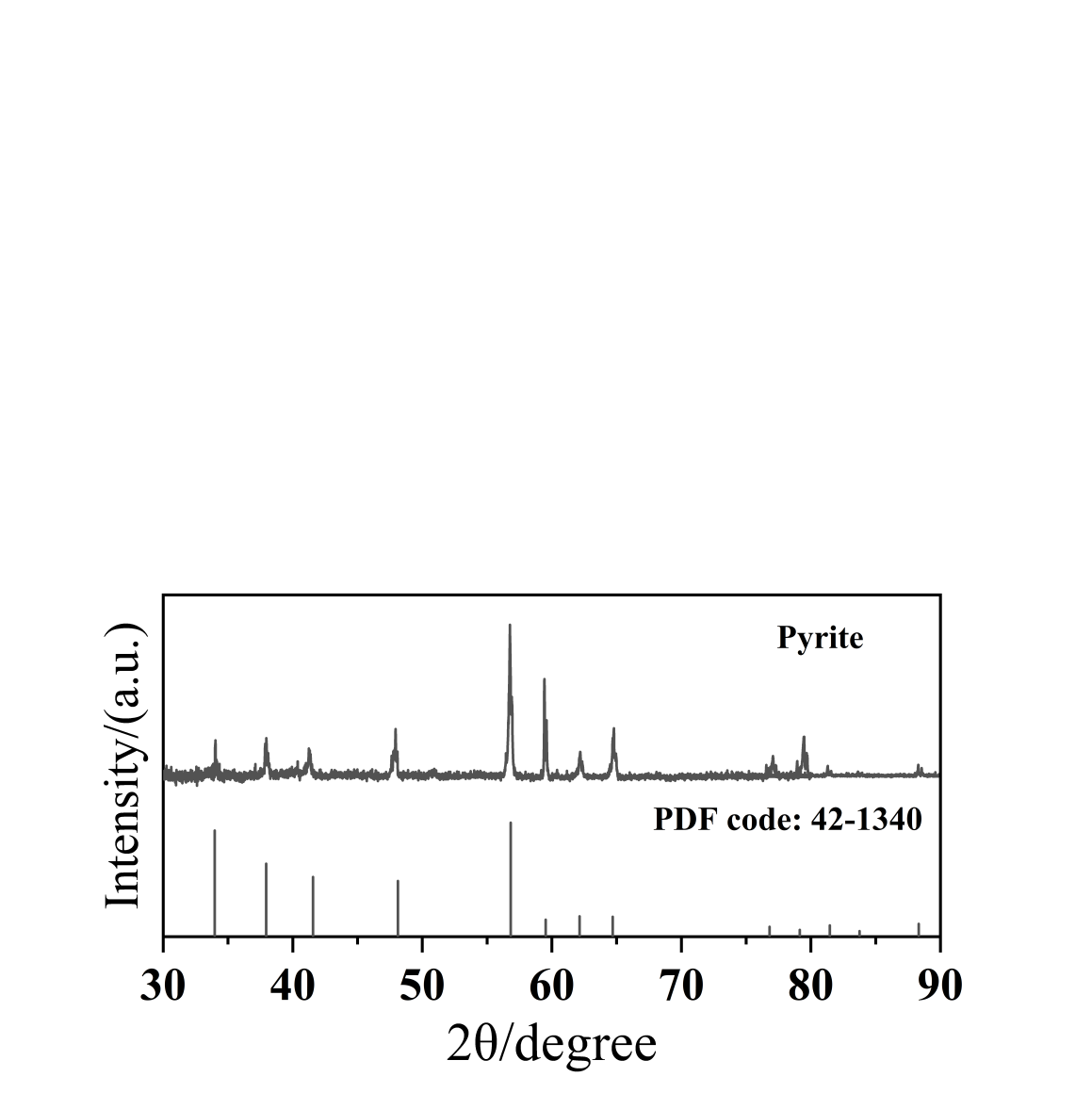


**Figure S1** XRD patterns of pyrite ore used in this study. The insert at the bottom demonstrates the standard XRD patterns of pyrite (PDF code: 42-1340).


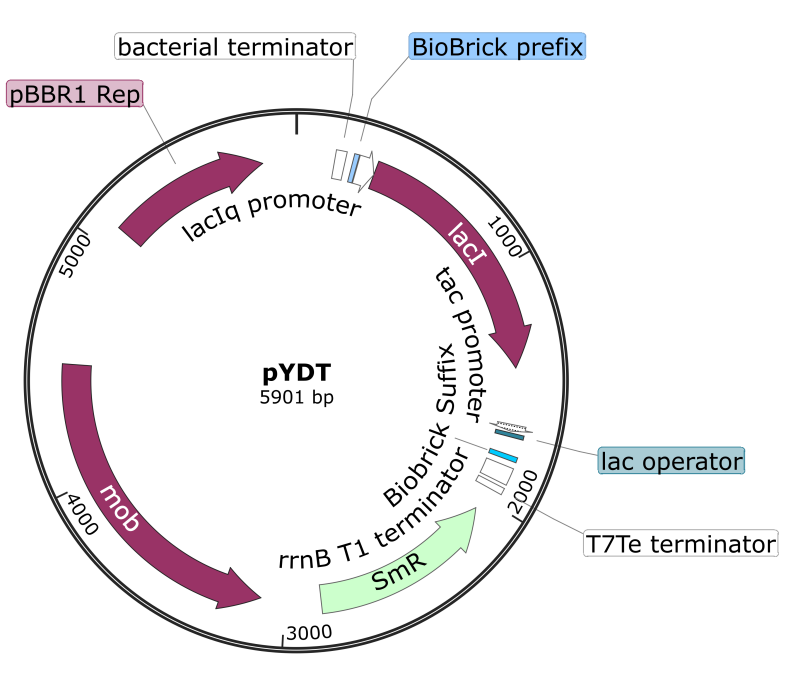


**Figure S2** Plasmid map of pYDT.


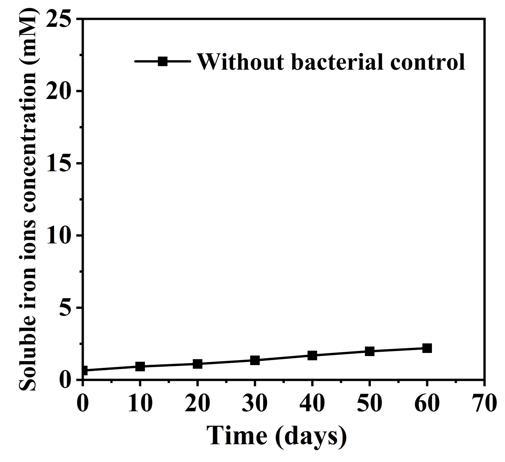


**Figure S3** Time course of iron ion release from pyrite (5 g in 50 mL suspension) in the sterile control group, with only a minimal amount of dissolved iron ions (~ 2.5 mM) released over 60 days.


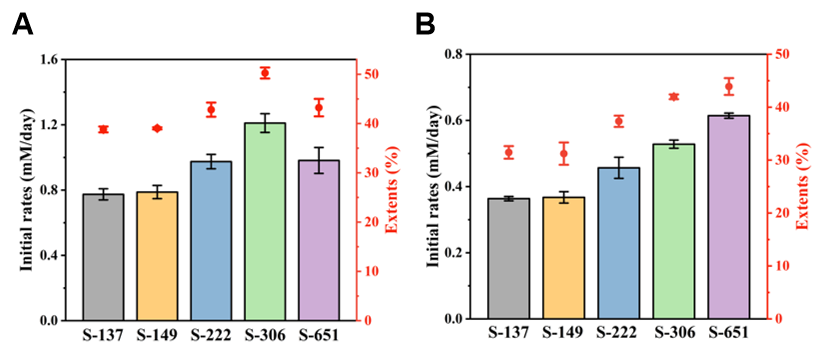


**Figure S4** Corresponding bioleaching parameters: iron release rates during the exponential phases (columns) and bioleaching extent (spots) under arsenic-free (A) and arsenic-stressed (B) conditions. Data are represented as mean values ± standard deviation (SD) (n = 3 independent replicates).


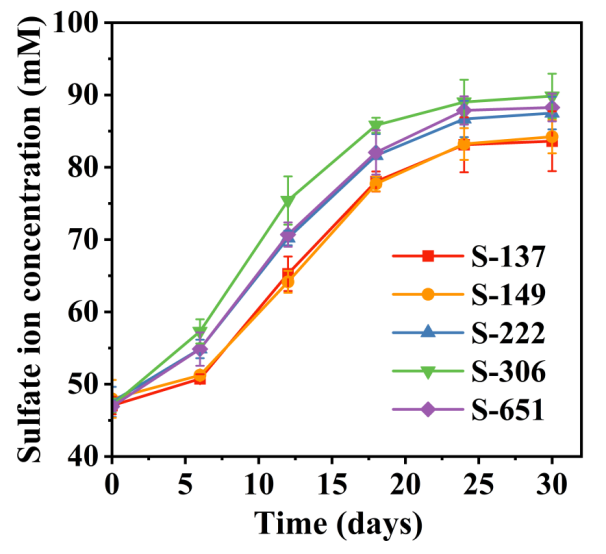


**Figure S5** Release kinetics of sulfate ions during microbial pyrite oxidation experiments by different strains under arsenic-free conditions (n = 3 independent samples).


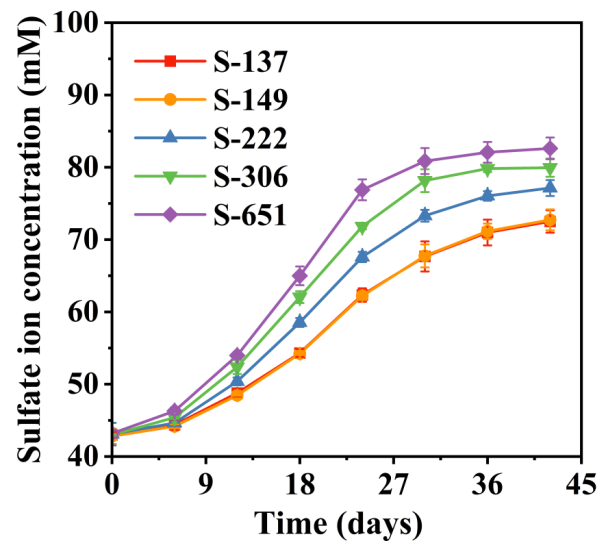


**Figure S****6** Release kinetics of sulfate ions during microbial pyrite oxidation experiments by different strains under arsenic-stressed conditions (n = 3 independent samples).


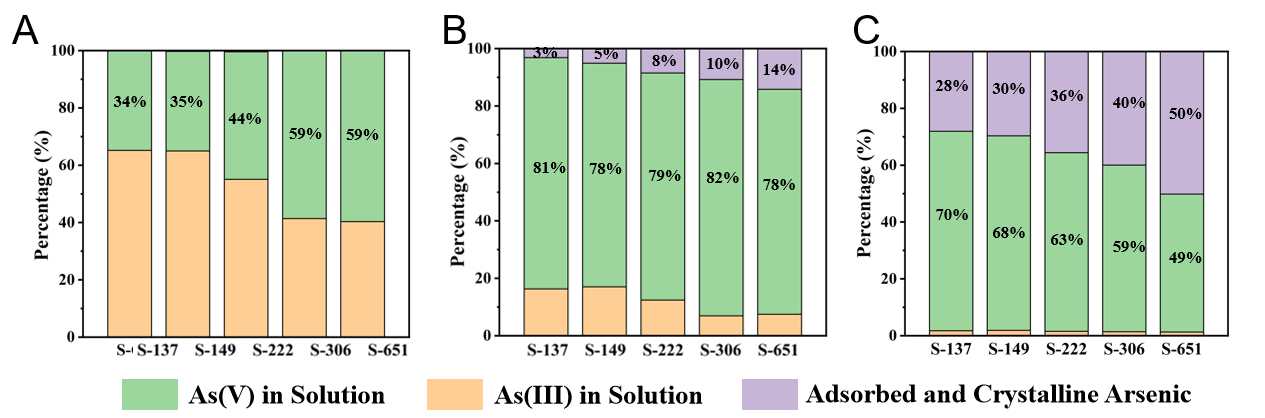


**Figure S7** Relative proportions of dissolved As(V) (green), and dissolved As(III) (yellow), and arsenic associated with solid products (purple) during arsenic-stressed bioleaching experiments: (A) day 6, (B) day 24, and (C) day 42.


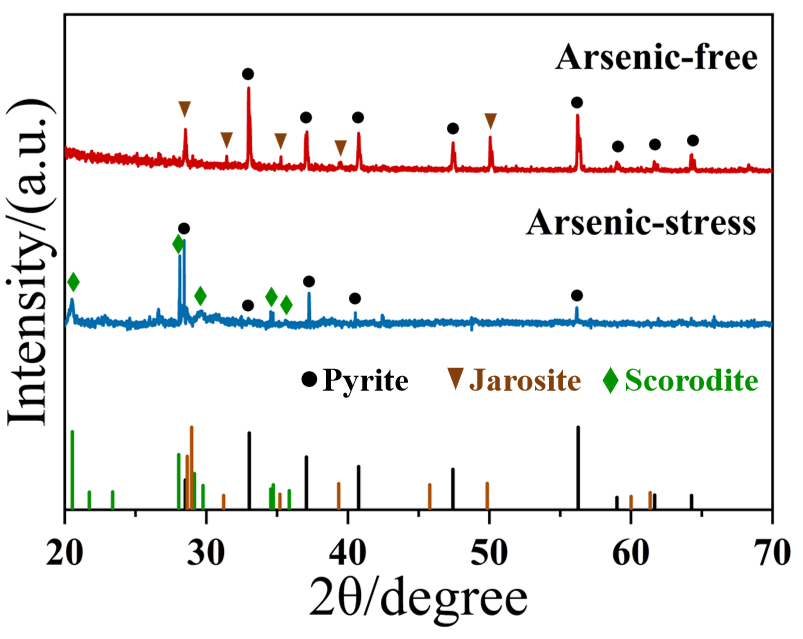


**Figure S8** XRD patterns of end-products from bioleaching experiments: top pattern (under arsenic-free conditions, regular powder XRD) and middle pattern (under arsenic-stressed conditions, synchrotron-based XRD). The insert at the bottom demonstrates the standard XRD patterns of pyrite (FeS₂, black spheres), jarosite (KFe₃(SO₄)₂(OH)₆, brown triangles), and scorodite ( FeAsO₄·2H₂O, green diamonds).


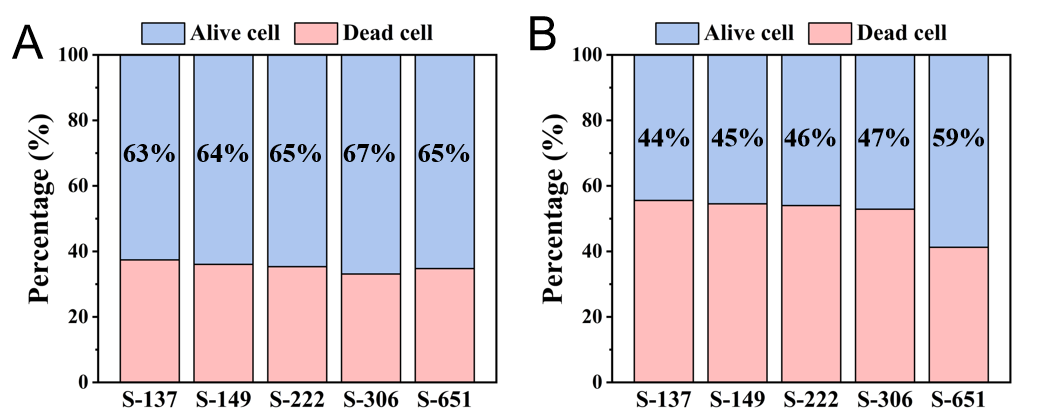


**Figure S9** Relative proportions of alive (blue) and dead (red) cells in biofilms of different strains formed on pyrite after bioleaching experiments under arsenic-free (A) and arsenic-stressed (B) conditions, derived from the corresponding representative CLSM images of biofilms (Fig. 2).


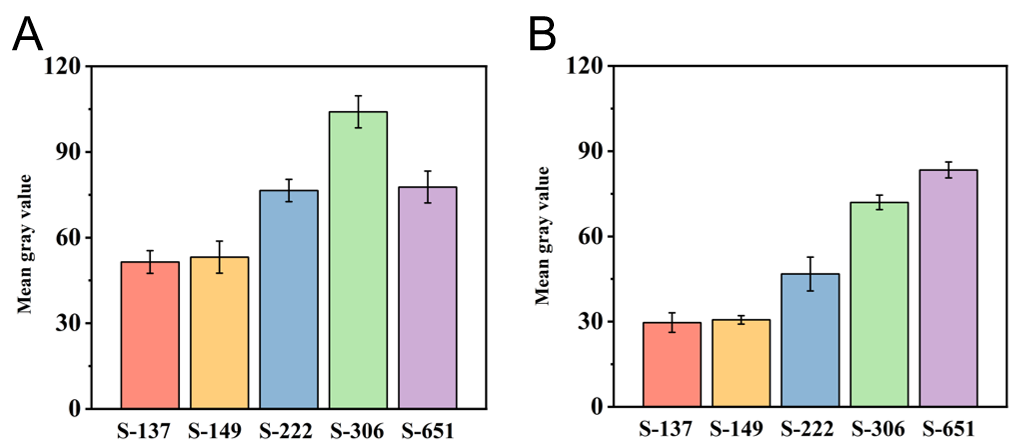


**Figure S10** Semi - quantitative analysis of biofilm biomass via mean gray value in live-cell CLSM images (Fig. 2) of the engineered strain biofilms formed on pyrite, under arsenic - free (A) and arsenic - stressed (B) conditions (ImageJ, n = 3).


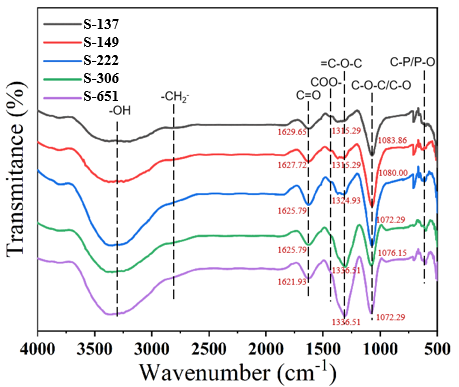


**Figure S11** FTIR spectra of biofilms from different strains on pyrite surfaces after arsenic-stressed bioleaching experiments.


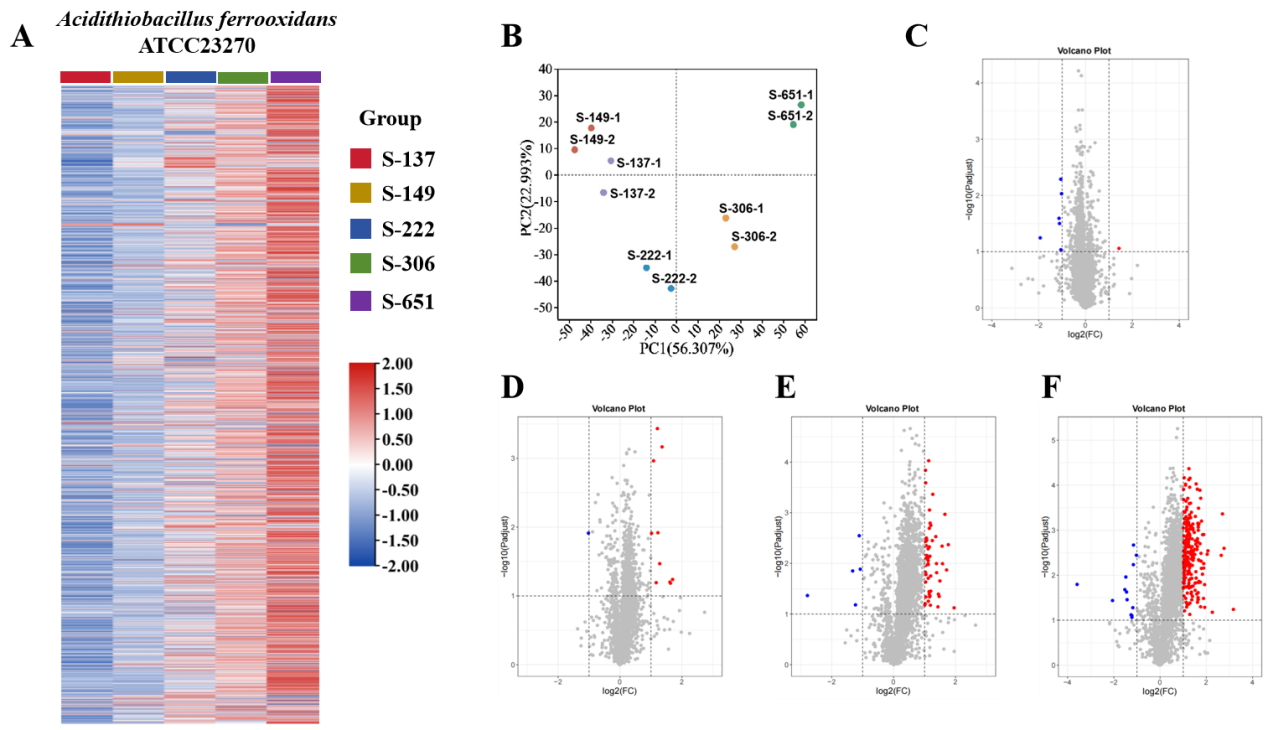


**Fig. S12** (A)Heatmap showing all genes in biofilm cells of strains S-137, S-149, S-222, S-306 and S-651. (B) Multidimensional scaling plot depicting distinct gene expression profiles of strains S-137 (control, empty vector), S-149, S-222, S-306, and S-651. (C–F) Volcano plots comparing different engineered strains to the control strain S-137: (C) S-149, (D) S-222, (E) S-306, and (F) S-651. All cells were collected after arsenic-stressed experiments.


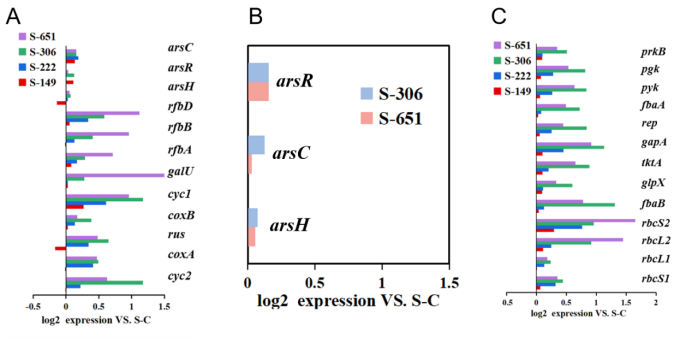


**Figure S13** Expression levels of arsenic resistance - encoding genes (*arsR*, *arsC*, and *arsH*) in engineered strains (S – 306 and S - 651) relative to control strain S - 137, showing no significant differences.


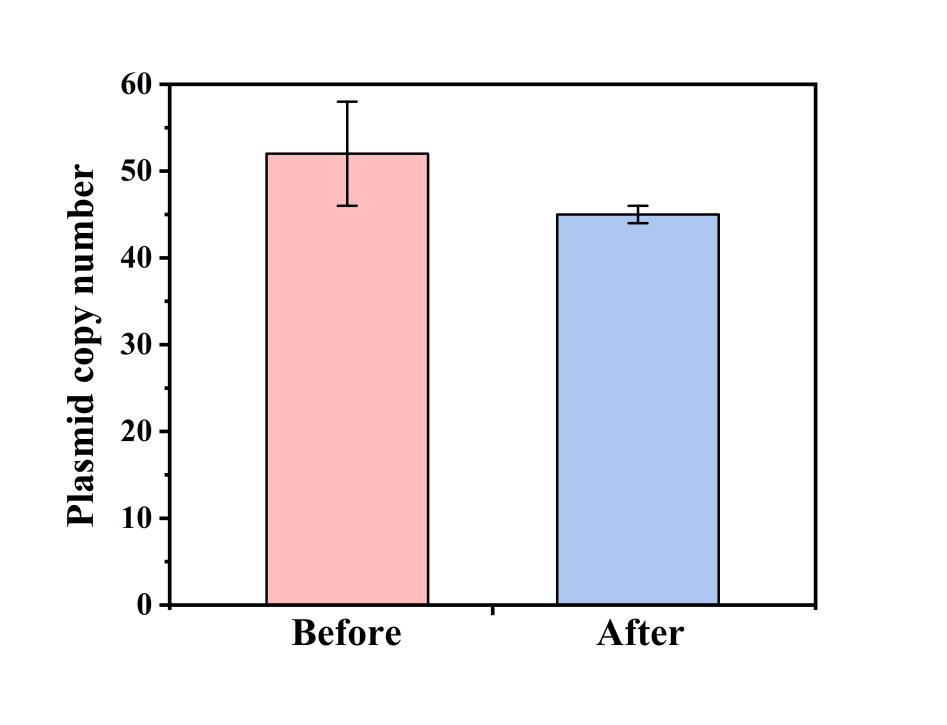


**Figure S14** The copy number of the pYDT vector before and after the bioleaching experiment lasting 45 days without antibiotics, respectively.

**Table S1 Strains and plasmids used in this study.**

| **Strain or plasmid** | **Description** | **Source** |
| --- | --- | --- |
| **Strains** | | |
| ***Escherichia coli*** | | |
| SM10 | Routine cloning host | Lab stock |
| ***Acidithiobacillus ferrooxidans* ATCC 23270** | |  |
| *A. ferrooxidans* | Wild type (WT) | Lab stock |
| S-137 | WT *A. ferrooxidans* carrying the empty vector pYDT | This study |
| S-149 | WT *A. ferrooxidans* carrying c-di-GMP synthase-encoding plasmid pYedQ | This study |
| S-222 | WT *A. ferrooxidans* carrying c-di-GMP synthase-encoding plasmid p1379 | This study |
| S-306 | WT *A. ferrooxidans* carrying c-di-GMP synthase-encoding plasmid p0053 | This study |
| S-651 | WT *A. ferrooxidan*s carrying c-di-GMP synthase-encoding plasmid p1373 | This study |
| **Plasmids** |  |  |
| pYDT | SmR; *oriV*(pBBR1), P*tac* | This study |
| pYedQ | Overexpress *yedQ* gene | This study |
| p1379 | Overexpress AFE-1379 gene | This study |
| p0053 | Overexpress AFE-0053 gene | This study |
| p1373 | Overexpress AFE-1373 gene | This study |

**Table S2 Primers used in this study.**

| **Primer** | **Sequence (5’ to 3’)** | **Usage** |
| --- | --- | --- |
| YedQ-F | TGCTCTAGAGCACAAAAGAGGAGAAATACTAGAGGTGCAGCACGAGACAAAAATGGAGAACCAGAGCTGGCT | Clone *yedQ* gene |
| YedQ-R | CGGCCGCTGCAGGTGCA | Clone *yedQ* gene |
| 1379-F | TGCTCTAGAGCACAAAAGAGGAGAAATACTAGAGGTGCAGCACGAGACAAAAGTGATTCCGATTGATCGTGGGC | Clone *AFE-1379* gene |
| 1379-R | ACCACCTGCAGTGCATTAAGCCAATGAATGA | Clone *AFE-1379* gene |
| 0053-F | TGCTCTAGAGCACAAAAGAGGAGAAATACTAGAGGTGCAGCACGAGACAAAAATGTTCGGGGCGATTCTGC | Clone *AFE-0053* gene |
| 0053-R | CGAGATGGAAGCCCGTTGATGGGACTAGTCC | Clone *AFE-0053* gene |
| 1373-F | TGCTCTAGAGCACAAAAGAGGAGAAATACTAGAGGTGCAGCACGAGACAAAAATGTTACAACAACCCACTCTGAACG | Clone *AFE-1373* gene |
| 1373-R | ACCAAAACTTGACCCATCGTGATGCACTGCAGTGCA | Clone *AFE-1373* gene |
| YD-F | GCCTCAGGCATTTGAGAAGCACA | pYDT sequence |
| YD-R2 | TTGACGAGTTCTTCTGAGCG | pYDT sequence |
| AlaS-F | CCATTGCGGATCTTGCAGC | *alaS*-qPCR |
| AlaS-R | CGGGCGATATCAGCGGTT | *alaS*-qPCR |
| GalU-F | GGGCTTTTCGACGATGCC | *galU*-qPCR |
| GalU-R | TCGGCGTCGAAGAAATCC | *galU*-qPCR |
| RfbA-F | ATCCGACTGCCACTTCCGTC | *rfbA*-qPCR |
| RfbA-R | AGTTCGATGCCGACCACCA | *rfbA*-qPCR |
| RfbB-F | CCGGCAATGGTCTTCCACA | *rfbB*-qPCR |
| RfbB-R | TCCCGAGAAACTGATTCCCC | *rfbB*-qPCR |
| Cyc2-F | GGGTAACCAGCACCTGCTGA | *cyc2*-qPCR |
| Cyc2-R | GAATACCACACCGGATTGGC | *cyc2*-qPCR |
| Rus-F | GACCGAATCCCTTGTTGGTG | *rus*-qPCR |
| Rus-R | CTCCCGGGATTTCCATTTC | *rus*-qPCR |
| CoxA-F | GAATCTCCAGCCATATGGCAA | *coxA*-qPCR |
| CoxA-R | CATTTTTACACCGGCCCCAC | *coxA*-qPCR |

**Table S3** **Components and their concentrations in 9K Medium (pH adjusted to 2.0 ± 0.2 with 3 M sulfuric acid)**

| **Component** | **Concentration** |
| --- | --- |
| (NH_4_)_2_SO_4_ | 3 g/L |
| K_2_HPO_4_ | 0.5 g/L |
| MgSO_4_∙7H_2_O | 0.5 g/L |
| KCl | 0.1 g/L |
| Ca(NO_3_)_2_ | 0.01 g/L |

**References：**

1. Gelder LD, Williams JJ, Ponciano JM, Sota M, Top EM. 2008. Adaptive plasmid evolution results in host-range expansion of a broad-host-range plasmid. Genetics 178:2179-90.

2. Lee C, Kim J, Shin SG, Hwang S. 2006. Absolute and relative QPCR quantification of plasmid copy number in *Escherichia coli*. Journal of Biotechnology 123:273-280.

3. Stookey LL. 1970. Ferrozine---a new spectrophotometric reagent for iron. Analytical Chemistry 42:779-781.

4. Zhong C, Ren Y, Guo YY, Lu A, Liu J. 2024. Photoelectron-promoted sulfate reduction for heavy metal removal without organic carbon addition. Environmental Science & Technology 58:21680-21691.

5. Cai P, Huang Q, Walker SL. 2013. Deposition and survival of *Escherichia coli* O157:H7 on clay minerals in a parallel plate flow system. Environmental Science & Technology 47:1896-1903.

6. Zhang J, Zhou F, Liu Y, Huang F, Zhang C. 2020. Effect of extracellular polymeric substances on arsenic accumulation in *Chlorella pyrenoidosa*. Science of The Total Environment 704:135368.
